# The MEK1/2-IRF4 axis fosters T follicular helper cell differentiationand antitumor humoral immune response

**DOI:** 10.1186/s12967-026-08023-2

**Published:** 2026-03-16

**Authors:** Shuan Ran, Song Wang, Ran Li, Longyong Lai, Jizhang Yu, Xi Zhang, Yuan Li, Weicong Ye, Junjie Zong, Xiaohan Li, Yanglin Hao, Jiulu Zhao, Zilong Luo, Han Zhang, Kexiao Zheng, Pinyan Huang, Wang Zhan, Zifeng Zou, Yanqiang Zou, Jikai Cui, Jie Wu, Jiahong Xia

**Affiliations:** 1 Department of Cardiovascular Surgery, Union Hospital, Tongji Medical College, Huazhong University of Science and Technology, Wuhan, China; 2 Center for Translational Medicine, Union Hospital, Tongji Medical College, Huazhong University of Science and Technology, Wuhan, China; 3 Key Laboratory of Organ Transplantation, NHC Key Laboratory of Organ Transplantation; Ministry of Education; , Chinese Academy of Medical Sciences, Wuhan, China

**Keywords:** Antitumor immunotherapy, Humoral immunity, T follicular helper cells, Mitogen-activated protein kinase kinase 1/2, Interferon regulatory factor 4

## Abstract

**Background:**

Emerging evidence has underscored the significance of antitumor humoral immunity, which is associated with the development of T follicular helper (Tfh) cell. However, the molecular mechanisms underlying Tfh differentiation in antitumor immunity remains poorly understood.

**Methods:**

We analyzed publicly available RNA-seq data across various representative immune models to identify signature pathways of Tfh cells. Subsequently, we generated T-cell-specific *Mek*1/2 knockout mice and investigated the functional phenotypes through Tfh cell polarization, adoptive transfer, and flow cytometry assays. Next, we employed multi-omics sequencing and overexpression experiments to delineate the underlying mechanisms of cellular phenotypes. Finally, by integrated analysis of scRNA-seq datasets combined with MEK inhibitor intervention studies, we elucidated the anti-tumor humoral responses of MEK inhibitors in both murine tumor models and TCGA-SKCM patient cohort.

**Results:**

Mitogen-activated protein kinase kinase 1/2 (MEK1/2) signaling was identified as a potential regulator of Tfh cells and antitumor humoral response. Codeletion or pharmacological inhibition of MEK1/2 effectively promoted Tfh cell development in vitro and in vivo. Mechanistically, ATAC-seq and RNA-seq integrative analysis revealed interferon regulatory factor 4 (IRF4)-dependent epigenetic modulation of characteristic Tfh cell genes, thereby driving Tfh differentiation. Overexpression of *Irf4* counteracted *Mek*1/2 ablation-mediated Tfh cell generation. Moreover, MEK1/2 inhibitor therapy enhanced Tfh infiltration coupled with humoral immune responses in murine melanoma model and was correlated with favorable clinical prognosis in melanoma patients.

**Conclusions:**

Our study revealed the MEK1/2-IRF4 axis potentiates Tfh development to enhance the antitumor humoral response. These findings may provide an unprecedented strategy to improve antitumor immunotherapy efficacy by harnessing humoral immune responses.

**Supplementary Information:**

The online version contains supplementary material available at 10.1186/s12967-026-08023-2.

## Introduction

T follicular helper (Tfh) cells specialize in orchestrating B-cell responses and are considered pivotal mediators bridging cellular immune defenses and antibody-driven humoral responses [1]. Extensive research has demonstrated that Tfh cells play critical roles in the initiation and progression of diverse immunological processes, including autoimmunity [2], infectious diseases [3], allergic disorders [4], and tumor immunity [5]. During Tfh cell ontogeny, elevated CXCR5 expression enables activated T-cell migration to the follicles [6], and their interaction with B cells contributes to the upregulation of the lineage-specific transcription factor BCL6 [7], which drives the Tfh cell program. The further development and function of Tfh cells are regulated by numerous other transcription factors (TFs), such as TCF1 [3], ASCL2 [8], BATF [9, 10], TOX2 [11] and c-MAF [12]. In addition, costimulatory molecules [13] and T-cell antigen receptor (TCR) signaling also modulate Tfh cell generation [14]. Despite these advances, unraveling the regulatory mechanisms governing Tfh cell differentiation remains a persistent challenge in immunology. Emerging evidence has highlighted the role of paired reciprocal antagonists in orchestrating the balance of Tfh cell versus non-Tfh cell differentiation fates, including the TF BCL6 and BLIMP1 [15], IL-6/IL-21-STAT3 [7, 16, 17] and IL-2-STAT5 signaling pathways [18–20]. More recently, Read et al. revealed that Aiolos represses CD4^+^ T-cell cytotoxic programming but potentiates the Tfh transcriptional profile and increases IL-2 sensitivity [21]. Notably, Krishnamoorthy et al. reported that, in a dose-dependent manner, IRF4 targets divergent DNA motifs to control Tfh and Teff cell gene programs [14]. However, whether additional signaling axes exist to regulate the fate bifurcation of Tfh versus non-Tfh cells remains much less clear.

Mitogen-activated protein kinase kinase 1/2 (MEK1/2), identified as the ‘gatekeeper’ of ERK1/2 activity [22], is rapidly activated downstream of initial TCR signaling to control T-cell positive selection, activation, proliferation, differentiation and survival [23–26]. Notably, pharmacologic inhibition of MEK preferentially blocks the expression of a series of important activated and functional markers in CD4^+^ T cells in vitro [27]. Similarly, Shindo et al. reported that MEK inhibitors selectively suppress alloreactive CD4^+^ T cells during murine graft-versus-host disease (GVHD) in vivo [28, 29]. In addition, Verma et al. reported that MEK1/2 inhibition generates a functional stem-like CD8^+^ T-cell phenotype with potent antitumor effects [30]. Moreover, Liu et al. revealed that the TCR-triggered MEK/ERK signaling pathway, which activates SMAD4, is critical for regulating the cytotoxic function of CD8^+^ T cells [31]. On the other hand, interferon regulatory factor 4 (IRF4), which is generally described as a central determinant of T-cell activation and development, exerts broad effects on T helper cell differentiation by regulating lineage-specific molecules [32]. Notably, Krishnamoorthy et al. demonstrated that the IRF4 gene regulatory module functions as a read-write integrator to dynamically coordinate effector CD4^+^ T-cell and Tfh cell development [14]. Similarly, our previous work revealed that ablation of IRF4 promotes transplant acceptance by driving allogenic CD4^+^ T-cell dysfunction [33]. Moreover, Man et al. reported that IRF4 promotes CD8^+^ T-cell exhaustion and limits the development of memory-like CD8^+^ T cells during chronic infection [34]. However, whether MEK1/2 and IRF4 regulate Tfh cell differentiation remains unreported and is worthy of further investigation.

In recent decades, emerging evidence has emphasized the importance of antitumor humoral immunity, characterized by Tfh-cell-driven B-cell responses, in various solid tumors [35–38]. For example, Gallo et al. initially identified the infiltration of CXCL13-producing CD4^+^ Tfh cells in tertiary lymphoid structures, which was correlated with a favorable clinical prognosis in breast cancer patients [39, 40]. Later, this group further revealed that functional Th1-oriented Tfh cells that infiltrate human breast cancer cells facilitate effective adaptive immunity [41]. In addition, Chaurio and colleagues demonstrated that TGF-β-mediated regulation of the genomic organizer Satb1 governs the differentiation of Tfh cells, resulting in the assembly of intratumoral tertiary lymphoid structures (TLSs) [42]. More recently, Fitzsimons et al. established a comprehensive pancancer single-cell RNA-seq landscape of intratumoral B and plasma cells [36], offering a reliable resource for further exploration. Ke et al. also provide insights into the colorectal cancer(CRC) immune macroenvironment, particularly revealing that tertiary lymphoid structures (TLS) presence correlates with patient survival and therapeutic efficacy of immune checkpoint inhibitors (ICI) [43]. However, one of the main challenges in investigating this problem is the extensive heterogeneity of components within the tumor immune microenvironment (TME), especially with respect to B-T-cell crosstalk [44, 45]. Small molecule inhibitor-based targeted therapy has proven to be a promising strategy for treating malignancies harboring oncogenic driver mutations. Among these, highly selective MEK1/2 inhibitors can effectively induce regression of melanoma bearing aberrant RAS‒MAPK pathway-activating mutations but seldom eradicate tumors [46, 47]. To address this limitation, most studies have focused on combination strategies involving small molecule inhibitors with various antitumor therapies [48–50]. However, little attention has been given to the influence of MEK inhibition in tumor-infiltrating lymphocytes (TILs) on potential augmented antitumor effects. Thus, further exploration of how small-molecule-targeted therapies modulate humoral immunity may improve the efficacy of cancer immunotherapies.

In this study, we revealed that the MEK1/2-IRF4 axis potentiates Tfh development and germinal center (GC) B-cell responses to enhance antitumor humoral immunity. Through bioinformatics analysis of Tfh cells in multiple immune models, we identified MEK1/2 signaling as a potential regulator of Tfh cells in response to immunization. Furthermore, in vivo and in vitro experiments confirmed that T-cell-specific *Mek1/2* ablation potentiated Tfh differentiation in a cell-intrinsic manner. Mechanistically, through integrative multiomics analysis, we revealed that IRF4-dependent epigenetic modulation of characteristic Tfh cell genes drives Tfh differentiation. More importantly, MEK1/2 inhibitor therapy resulted in enhanced humoral immune responses in a murine melanoma model and was correlated with a favorable clinical prognosis in melanoma patients. Taken together, these data indicate that the MEK1/2-IRF4 axis is a potential therapeutic target for improving antitumor humoral immunity.

## Materials and methods:

### Mice:

*Cd4-Cre*, OT-II TCR transgenic, *Rag1*^-/-^, and CD45.1 (Ptprca Pepcb/BoyJ) mice were purchased from the Jackson Laboratory (USA). Male C57BL/6J (B6) mice (aged 6–12 weeks) were obtained from Charles River (Beijing, China). *Map2k1* and *Map2k2* flox-bearing mice were generated by Shanghai Model Organisms (Shanghai, China) on a C57BL/6 background. Specifically, *Map2k1* flox mice were generated via transcription activator-like effector nuclease-mediated insertion of loxP sites in the *Map2k1* gene, whereas *Map2k2* flox mice were generated via CRISPR/Cas9-mediated insertion of loxP sites in the *Map2k2* gene. *Map2k1*^fl/fl^ and *Map2k2*^fl/fl^ mice were crossed with *Cd4-Cre* mice to generate T-cell-specific *Map2k1* and *Map2k2* double-gene knockout (DKO) mice. *Map2k1* and *Map2k2* genotyping was performed on tail DNA via PCR via a one-step mouse genotyping kit (Vazyme, PD101-01). The primers used were as follows: 5-TGGCCTCGAACTCAGAAATCCA-3; 5-TAGAAGGAACGGCACAATACAAAC-3; 5-CCGGTGCTGCTGAGGAAGGA-3; and 5-CCAAGAACCAGGCCCAAGAGG-3. OT-II *Cd4-Cre*^+^*Map2k1*^fl/fl^ and *Map2k2*^fl/fl^ mice (on a C57BL/6 background) were generated by crossing *Map2k1*^fl/fl^ and *Map2k2*^fl/fl^ mice with OT-II (C57BL/6-Tg (TcraTcrb)425Cbn/Crl, Charles River) mice. *Cd4-Cre*^+^*Irf4*^fl/fl^ knockout mice were were derived from our previous research [33]. All the mice were maintained in the special pathogen-free animal facility of Huazhong University of Science and Technology. All animal experiments in this study were approved by the Institutional Animal Care and Use Committee of Huazhong University of Science and Technology.

### Reagents and antibodies

The fluorophore-conjugated antibodies used for flow cytometry were as follows: FITC anti-mouse CD62L Antibody (MEL-14, #104406), PerCP/Cyanine5.5 anti-mouse/human CD44 Antibody (IM7, #103032), PE/Cyanine7 anti-mouse CD279 (PD-1) Antibody (29F0.1A12, #135216), Brilliant Violet 421™ anti-mouse CD185 (CXCR5) Antibody (L138D7, #145512), Alexa Fluor® 647 anti-mouse/human Bcl-6 Antibody (IG191E/A8, # 648,305), PE anti-mouse CD279 (PD-1) Antibody (29F0.1A12, #135205), APC anti-mouse CD185 (CXCR5) Antibody (L138D7, #145506), PE anti-mouse CD4 Antibody (GK1.5# 100,408), PE/Cyanine7 anti-mouse CD8a Antibody (53–6.7, # 100,722), FITC anti-mouse TCR-β chain Antibody (H57-597,#109206), PE anti-mouse CD95 (Fas) Antibody(SA367H8,#152608), PE/Cyanine7 anti-mouse CD138 (Syndecan-1) Antibody (281–2,#142513), PE/Cyanine7 anti-mouse CD45.2 Antibody (104,#109830), FITC anti-mouse TCRVα2 Antibody(B20.1, #127806), PE/Cyanine7 anti-mouse CD366 (Tim-3) Antibody (RMT3-23,#119715), PE Donkey anti-rabbit IgG Antibody (Poly4064,#406421) were from BioLegend; APC-Cy™7 Rat Anti-Mouse CD4 antibody (GK1.5,# 552,051), BV421 Mouse Anti-Bcl6 antibody (K112-91,# 563,363), Alexa Fluor® 647 Rat Anti-Mouse *T*- and B-Cell Activation Antigen antibody (GL7,#561529), PerCP-Cy™5.5 Rat Anti-Mouse CD45R/B220 antibody (RA3-6B2,#561101), Alexa Fluor® 647 Mouse Anti-TCF-7/TCF-1 (S33-966,#566693) were from BD Biosciences; PE-TOX Monoclonal Antibody (TXRX10, #2536035) were from invitrogen; Phospho-MEK1/2 (Ser221) (166F8) Rabbit mAb (PE Conjugate) (#16211S), Phospho-p44/42 MAPK (Erk1/2) (Thr202/Tyr204) (E10) Mouse mAb (Alexa Fluor® 647 Conjugate)(#4375S), Acetyl-Histone H3 (Lys27) (D5E4) Rabbit Monoclonal Antibody(#8173), Acetyl-Histone H3 (Lys9) (C5B11) Rabbit Monoclonal Antibody (#9649), Tri-Methyl-Histone H3 (Lys4) (C42D8) Rabbit Monoclonal Antibody (#9751),Tri-Methyl-Histone H3 (Lys36) (D5A7) Rabbit Monoclonal Antibody (#4909) were from cell signaling technology;

### Immune model

To induce Tfh formation and the GC response, chicken ovalbumin (OVA, 1 mg/mL, Sigma) was emulsified in complete Freund’s adjuvant (CFA, 2 mg/mL) at a 1:1 ratio. Then, 50 μL of emulsion was subcutaneously and symmetrically injected at the tail base of each mouse. On day 7 after immunization, the recipient mice were sacrificed, and the draining lymph nodes were analyzed individually. For sheep red blood cell (SRBC, Veterinary Services, IMVS) immunization, the mice were intraperitoneally injected with 1 × 10^9^ SRBCs (suspended in 0.2 ml of PBS) to induce a T-cell-dependent GC response, and the splenic cells were collected on day 7 and then analyzed via flow cytometry.

### Tumor model and inhibitor treatment

For tumor model establishment, the mice were first shaved and subcutaneously inoculated with B16_OVA cells (1 × 10^6^ cells resuspended in 100 μl of PBS per mouse) in the right flank on day 0. The MEK inhibitor was subsequently administered via oral gavage from day 7 to day 15, with trametinib (GSK1120212, JTP-74057; MedChemExpress) dosed at 0.3 mg/kg/day in a vehicle of 10% DMSO/90% corn oil (v/v). The control groups received equivalent volumes of corn oil vehicle. On day 15 after inoculation, the recipient mice were sacrificed, and the draining lymph nodes were harvested and measured by FCM individually.

For the B-cell transfer assay, B cells were collected from spleens from naive C57BL/6 mice by negative enrichment using the mouse Pan B cell isolation kits from Miltenyi Biotech (#130–095-813). *Rag1*^-/-^ mice were firstly inoculated with B16_OVA cells (1 × 10^6^ cells resuspended in 100 μl of PBS per mouse) at day0 and were immunological reconstituted with 5 × 10^6^ OT-II T cells plus 5 × 10^6^ B cells at day5, followed by treated with trametinib (0.3 mg/kg/day; resuspended in 10% DMSO with 90% corn oil) or equivalent Corn oil by oral gavage daily from day5 to day20, tumor draining lymph nodes (dLNs) were harvested and measured by FCM at day12.

For the B-cell depletion assay, following intraperitoneal administration of 250 μg anti-mouse CD20 monoclonal antibody (clone MB20-11, catalog BE0356, Bio X Cell) or isotype control one day prior, C57BL/6 mice were inoculated with B16F10 cells (1 × 10^6^ cells resuspended in 100 μl of PBS per mouse) on day 0 and then underwent daily oral gavage with either trametinib (0.3 mg/kg/day; resuspended in 10% DMSO with 90% corn oil) or corn oil from day5 to day20.Tumors were regularly monitored every 2 days with a digital vernier caliper and tumor volumes were calculated using the equation: V= (length ×width2)/2.

### Isolation and polarization of CD4^+^ T cells

Single-cell suspensions were harvested from the spleens after being mechanically mashed, the samples were filtered through a 70-μm cell strainer, and erythrocytes were removed by using ACK lysing buffer (A1049201, Gibco). CD4^+^ T cells were subsequently isolated from single-cell suspensions via positive selection using the MojoSort™ Mouse CD4 Naïve T Cell Isolation Kit (#480039, Biolegend). The cell suspensions were washed twice with cold PBS supplemented with 2% fetal bovine serum (FBS).

In vitro Tfh polarization was conducted as previously described. In brief, 5 μg/mL anti-CD3 antibody (BD Biosciences, catalog 553,057) was mixed in PBS and coated in 96-well round-bottom tissue culture plates at 4 °C overnight. Then, naive CD4^+^ T cells were further activated with 1 μg/mL anti-CD28 (BD Biosciences; catalog 553,294), and medium was additional supplemented with anti-IL-2 (BD Biosciences, catalog554424; 10 μg/mL), and anti–IFN-γ (BD Biosciences, catalog 554,430; 10 μg/mL), and anti-IL-4 (BD Biosciences, catalog 554,385; 10 μg/mL), and anti-TGF-β (Thermo Fisher Scientific, catalog 16–9243-85; 10 μg/mL) plus cytokines IL-6 (PeproTech, 216–16; 20 ng/mL). For the inhibition experiments, 0.2% DMSO or 100 nM trametinib was added to the indicated groups. After being cultured for 3 days, the cells were collected and analyzed via flow cytometry coupled with RNA sequencing.Th cells were polarized from sorted naïve CD4^+^ T cells activated with 1 μg/ml anti-CD3/anti-CD28 for 3d in RPMI 1640 medium (Gibco) supplemented with different cytokine mixes. For Th1 polarization, 5 ng/ml IL-12 (R&D Systems), 3 μg/ml anti–IL-4 (BioXcell), and 20 U/ml rhIL-2 (PeproTech) were used. For Th17 polarization, 20 ng/ml IL-6, 20 ng/ml IL-23 (R&D Systems), 10 ng/ml IL-1β (BioLegend) and 1 ng/ml rhTGF-β1 (BioLegend) were used. After 3 days, cells were collected and analyzed via FCM.

### Cell culture

For in vitro T-cell culture, sorted CD4^+^ T cells were resuspended in RPMI 1640 medium (Gibco) supplemented with 10% FBS (Gibco), penicillin‒streptomycin (15070063; Gibco), hydroxyethylpiperazine ethane sulfonic acid (HEPES; H1090, Solarbio), glutaMAX (35050061; Gibco), and 2-mercaptoethanol (21985023; Gibco) and then cultured at 37 °C in a humidified atmosphere with 5% CO^2^. B16_OVA and B16F10 cells were gifted from Dr. Xiangliang Yang (Huazhong University of Science and Technology, China) and were cultured in RPMI 1640 medium (PM150110, Procell) supplemented with 10% fetal bovine serum (10100139, Gibco) and 1% antibiotics (BL505A, Biosharp) at 37 °C in a humidified 5% CO2 incubator.

### Adoptive T-cell transfer

For the in vivo OVA/CFA immunization experiment, 1 × 10^6^ CD45.2^+^ WT or *Mek*1/2-tKO OT-II T cells were adoptively transferred into CD45.1^+^ B6 recipient mice by intravenous tail vein injection one day ahead, then recipient mice were subjected to OVA/CFA immunization. On day 7 after immunization, the Tfh cell counts were further assessed by flow cytometry. RNA-seq and ATAC-seq analyses were performed on the transferred CD45.2^+^ WT or *Mek*1/2-tKO OT-II T cells from the dLNs of the recipient mice.

For the *Irf4* overexpression experiment, we sorted splenic naïve CD4^+^ T cells from CD45.2^+^*Mek*1/2-tKO OT-II^+^ B6 mice and enforced the expression of *Irf4* via retroviral transduction with empty or RV-*Irf4*-GFP^+^ vectors. Subsequently, 1 million infected cells were transferred to CD45.1^+^B6 mice on day 1 via intravenous tail vein injection, and then the recipient mice were subjected to OVA/CFA immunization on day 0, followed by FCM analyses of CD45.2^+^ OT-II T cells in dLNs on day 7.

### Retroviral transduction of IRF4

Retroviral transduction of IRF4 was performed as previously described [33]. Retroviral supernatant was produced by transfecting plat-E cells with the IRF4–GFP or empty control (Ctrl)–GFP retroviral vector according to the manufacturer’s instructions (pMYs–IRES–eGFP, Cell Biolabs). For retroviral transduction, naïve CD4^+^ T cells were isolated from the spleens of CD45.2^+^*Mek*1/2-tKO OT-II^+^ B6 mice by using the MojoSort™ Mouse CD4 Naïve T-Cell Isolation Kit (#480039, Biolegend). These sorted CD4^+^ T cells were first activated in vitro for 24 hours with anti-CD3/CD28 beads (11452D, Thermo Fisher) and then incubated with freshly prepared viral supernatants by centrifugation at 780 × g and 32 °C for 2 hours in the presence of 0.8 mg/ml polybrene (Sigma‒Aldrich). Following centrifugation, the transduced cells were cultured at 32 °C for 6 hours and subsequently cultured in complete RPMI 1640 medium supplemented with 100 U/mL recombinant murine IL-2 (P04351, PeproTech) at 37 °C for an additional 72 hours. Finally, 1 × 10^6^ sorted CD45.2^+^*Mek*1/2-tKO OT-II^+^ cells expressing IRF4–GFP or Ctrl–GFP were adoptively transferred into CD45.1^+^ B6 mice one day before OVA/CFA immunization. On day 7, flow cytometry analysis was performed for CD45.2^+^ OT-II^+^ T cells in dLNs.

### Flow cytometry

Spleens, thymuses and peripheral draining lymph nodes (dLNs) were harvested, mechanically mashed, and then passed through a 70-μm cell strainer to obtain single-cell suspensions. Subsequently, red blood cells (RBCs) were lysed in ACK lysis buffer (A1049201, Gibco). The cell suspensions were washed twice with cold PBS containing 2% fetal bovine serum and centrifuged (2000 rpm, 3 min). Before staining, the cells were blocked with an anti-CD16/32 antibody (BioLegend; clone 93) for 20 min at 4 °C. Dead cells were excluded by incubation with a Zombie Aqua/NIR/Green Fixable Viability Kit (BioLegend).

For surface molecule staining, the cells were resuspended in a fluorochrome-conjugated antibody cocktail and incubated in the dark at 4 °C for 30 minutes. For intracellular staining of transcription factors, the cells were fixed and permeabilized with an intracellular/transcription factor staining kit (00–5523-00; eBiosciences) at 4 °C for 30 min and then washed with 1× permeabilization buffer, followed by staining with fluorochrome-labeled antibodies at room temperature for 1 hour according to the manufacturer’s instructions. For the detection of IRF4, the cells were first fixed and permeabilized as described above, followed by incubation with a mouse anti-IRF4 monoclonal antibody (sc-48338, Santa Cruz Biotechnology) for 60 min at 4 °C and subsequent staining with a PE/cyanine7 goat anti-mouse IgG antibody (Poly4053, BioLegend) for 30 min at 4 °C.

For intracellular staining of phospho-MEK1/2 and phospho-ERK1/2, the cells were restimulated with 1 μg/ml PMA and 1 μg/ml ionomycin for 15 min at 37 °C. After centrifugation and removal of the supernatant, the cells were resuspended in 100 µl of 4% formaldehyde per million cells and fixed for 15 min at room temperature. Then, the cells were permeabilized for 10 min on ice by slowly adding ice-cold 100% methanol to prechilled cells while gently vortexing to a final concentration of 90% methanol. Finally, the cells were stained with the AF647-conjugated phospho-p44/42 MAPK (Erk1/2) (Thr202/Tyr204) (E10) mouse mAb (#4375S; Cell Signaling Technology) or PE-conjugated phospho-MEK1/2 (Ser221) (166F8) rabbit mAb (#16211S; Cell Signaling Technology) for 1 hour in the dark at room temperature.

For indirect intracellular staining of H3K4me3, H3K36me3, H3K9ac and H3K27ac, cells were fixed and permeabilized as described above and then stained with Acetyl-Histone H3 (Lys27) (D5E4) Rabbit Monoclonal Antibody(Cell Signaling Technology, #8173), Acetyl-Histone H3 (Lys9) (C5B11) Rabbit Monoclonal Antibody (Cell Signaling Technology, #9649), Tri-Methyl-Histone H3 (Lys4) (C42D8) Rabbit Monoclonal Antibody (Cell Signaling Technology, #9751), and Tri-Methyl-Histone H3 (Lys36) (D5A7) Rabbit Monoclonal Antibody (Cell Signaling Technology, #4909) at 37 °C for 60 min, followed by staining with a PE donkey anti-rabbit IgG antibody (Biolegend, #406421) at 37 °C for 30 min.

After staining as described above, the cells were processed with an ID7000 flow cytometer (Sony) or an LSR Fortessa X-20 flow cytometer (BD Biosciences), and the data were analyzed with FlowJo v10 software (BD Biosciences).

### ELISA

On day 7 after OVA/CFA immunization or day 15 after B16_OVA inoculation, all the mice were euthanized, and the serum was collected. For the detection of OVA-specific IgG1 antibodies, an enzyme-linked immunosorbent assay was performed via an anti-ovalbumin IgG1 (mouse) ELISA kit (Cat#: 500,830, Cayman Chemical) according to the manufacturers’ protocol. Briefly, 100 μl of standard or diluted serum was added to each well of 96-well plates, which were subsequently incubated for 2 hours at room temperature. After the samples were fully rinsed 4 times with wash buffer, 100 μl of goat anti-mouse IgG1 HRP detection antibody working solution was added to each well, and the samples were incubated for 1 hour at room temperature. The samples were subsequently washed as described above, and 100 μl of TMB substrate solution was added to each well. After 30 minutes of TMB incubation at room temperature, 100 μl of HRP stop solution was added to each well. While blue wells turned yellow within 30 minutes, the optical density (OD) values were measured with a microplate reader at 450 nm.

### Chromatin immunoprecipitation (ChIP) assay

ChIP was performed using a ChIP Assay Kit (Cell Signaling Technology, #9003) according to the manufacturer’s instructions. Briefly, after In vitro Tfh polarization, WT or tKO CD4^+^ T cells were cross-linked using 1% formaldehyde for 10 min at 37 °C. Chromatin was enzymatically digested to yield DNA fragments of approximately 200–1000 bp. An aliquot corresponding to 1% of the chromatin was reserved as input prior to immunoprecipitation. Immunoprecipitation was carried out using a ChIP-grade H3K4me3 antibody (Cell Signaling Technology, #9751), with normal rabbit IgG (Cell Signaling Technology, #2729) serving as a negative control. Cross-links were reversed by NaCl- and Proteinase K–mediated decrosslinking, followed by column-based DNA purification. Purified DNA was subjected to real-time PCR to quantify the tri-methyl-H3K4 level on the gene promotor using specific primers. The ChIP-qPCR primers used in this study are list: Forward: 5’-GCATTTCGCACCTCGCCCTTCG-3’; Reverse: 5’-CTCAGGGCCGGCGTGAAGGCT-3’. The results were calculated and are expressed as the relative enrichment of H3K4me3 on the gene promotor.

### GEO dataset bioinformatics analysis

Published RNA-seq datasets of T follicular helper (Tfh) cells in different immune models were downloaded from the Gene Expression Omnibus (GEO) database, including five murine datasets (GSE144467, GSE183316, GSE40068, GSE21380, and GSE226341) and one human dataset (GSE58596). Differential expression was analyzed via the DESeq2 package (v1.40.1) or limma package (v3.56.2) of R software (v4.3.0). DEGs between Tfh and non-Tfh cells were identified by the criteria of absolute fold changes ≥ 1.5 and p-adjusted < 0.05, followed by downstream functional analysis. Pathway enrichment analysis was performed with the ‘enrichKEGG’ or ‘enrichGO’ function in clusterProfiler (v4.8.1.). The KEGG and GO collections were obtained from the Molecular Signatures Database (MSigDB). The core pathway signature for Tfh cells was generated by intersecting the lists of multiple differentially enriched KEGG pathways across 5 murine datasets, followed by visualization with UpSetR (v1.4.0). The fold enrichment of each shared pathway was evaluated by dividing the GeneRatio by the BgRatio and visualized with ggradar (v0.2). Principal component analysis (PCA) was performed with FactoMineR (v2.8), and the results were plotted by factoextra (v1.0.7). Chord plot of the GO enrichment results was created using GOplot (v1.0.2). The epigenetic features of the *Irf4* promotor locus with active chromatin marker H3K4me3 ChIP-Seq data (GSE52840) in Tfh cells and non-Tfh was visualized via the Integrative Genome Viewer (v2.13.1).

### Bulk RNA-seq

For bulk RNA sequencing, ex vivo-polarized WT or *Mek*1/2-tKO CD4^+^ T cells were resorted to viable cells on day 3. CD45.2^+^ WT or *Mek*1/2-tKO OT-II T cells in dLNs were isolated from recipient mice on day 7 after OVA/CFA immunization. Total RNA from the above sorted cells was extracted with TRIzol Reagent (Invitrogen, cat. No. 15,596,026) and used for RNA-seq library construction via the KCTM Stranded mRNA Library Prep Kit for Illumina® (Catalog No. DR08402, Wuhan Seqhealth Co., Ltd., China). The libraries were sequenced on a NovaSeq 6000 sequencer (Illumina) in paired-end read mode with the read length of 150 nucleotides. The raw data were first filtered by using Trimmomatic (v0.36) to remove low-quality reads and adaptor sequences. The clean reads were mapped to the GRCm38 genome via STRA software (v2.5.3a) with default parameters, and then uniquely mapped reads were converted to the gene expression matrix by featureCounts (Subread-1.5.1; Bioconductor).

The downstream analysis was carried out as described above. DEGs were identified by at least 1.5-fold change and adjusted *p* value of 0.05 using the DESeq2 package (v1.40.1). PCA was analysed with FactoMineR (v2.8) and visualized by factoextra (v1.0.7). The scatterplot was plotted by the “sp_scatterplot” function in the ImageGP package (v0.1.0), and the MA plot was created with ggplot2 (v3.4.2). To display the selected characteristic genes via a heatmap, we employed the pheatmap package (v1.0.12). For gene set enrichment analysis, the ‘GSEA’ function in clusterProfiler (v4.8.1) was performed based on the Tfh or non-Tfh upregulated signature gene set as previously described by Liu and then visualized via the ‘gseaNb’ function of GseaVis (v0.0.5). Gene Ontology (GO) enrichment for DEGs was analyzed with the “enrichGO” function and plotted by ggplot2 (v3.4.2) or the ‘radarchart’ function of Fmsb (v0.7.5).

### ATAC-seq

For ATAC sequencing, transferred CD45.2^+^ WT or *Mek*1/2-tKO OT-II T cells in dLNs were isolated from recipient mice on day 7 after OVA/CFA immunization. A total of 5 × 10^4^ sorted fresh cells were lysed, and the nuclei were collected by centrifugation at 500 × g for 5 min. Subsequently, standard transposition reactions and library construction were conducted with a TruePrep DNA Library Prep Kit V2 for Illumina (catalog no. TD501, Vazyme) according to the manufacturer’s instructions. After enrichment and quantification of the library products, they were sequenced on a NovaSeq 6000 sequencer (Illumina) with a PE150 model through SeqHealth Technology Co., Ltd. (Wuhan, China).

The raw data were first checked by FastQC (v0.11.5) and filtered via fastp (v0.23.1) to discard low-quality reads and adaptor sequences. The clean reads were aligned to the GRCm38 genome using Bowtie2 (v2.2.6). Sambamba (v0.7.1) was used to perform sam/bam format conversion and PCR duplicate reads removal. The insert length was calculated by the Collect Insert Size Metrics tools from Picard software (v2.8.2), and DeepTools (v2.4.1) was used to visualize the distribution of reads up- and downstream of the transcription start site (TSS). Peak calling was conducted using MACS2 software (v2.7.1), after which a consensus peak set was obtained. To assess differentially accessible chromatin regions, the DiffBind package (v3.10.1) was used in R software (v4.3.0) with the ‘dba.analyze’ function. The ChIPseeker package (v1.36.0) was used to annotate the above differential sites with associated genes and was used for downstream analysis.

### ATAC-RNA integrative analysis

For the combined analysis of RNA-seq and ATAC-seq data, peak log_2_ (FC) scores from ATAC-seq differential analysis and gene log_2_ (FC) scores from RNA-seq differential analysis were tabulated and gene-matched. The ‘cor.test’ function of the stats package (v4.3.0) was subsequently applied to evaluate the correlation of the transcriptional and epigenetic changes. Scatterplots were generated using ggplot2 (v3.4.2). Furthermore, motif enrichment analysis was performed with Homer (v 4.10) based on known motif datasets. The enrichment analysis of the IRF4 binding motifs was conducted according to the methods of Krishnamoorthy [14]. FIMO [51] was applied to search for instances of IRF4 binding motifs. The proportion of chromatin-accessible regions (ChARs) in a subcategory that contained a motif versus that proportion in all ChARs was subsequently calculated, and the subcategory-specific enrichment of each motif was finally obtained. Heatmaps were generated via the enrichment ratio (Log_2_) using the pheatmap package (v1.0.12). Finally, the chromatin accessibility of Tfh and non-Tfh cell-characteristic genes was visualized via the Integrative Genome Viewer (v2.13.1).

### scRNA-seq

Single-cell RNA-seq data of tumor-infiltrating CD45^+^ immune cells derived from melanoma patients were retrieved from the GEO database (GSE177901, GSE215120). The preprocessed single cell-gene expression matrices were imported into R software (v4.3.0) and analyzed via the Seurat package (v4.3.0.1). Cells with unique gene counts less than 500 or more than 5000 and mitochondrial percentage above 10% were excluded from downstream analyses. The matrix was subsequently normalized and transformed via the ‘NormalizeData’ function. Two thousand highly variable genes were identified using the ‘FindVariableFeatures’ function with the ‘vst’ method. Next, the scale and center of the gene expression matrix were determined via the ‘ScaleData’ function, followed by linear dimensional reduction via the ‘RunPCA’ function. To remove batch effects, Harmony (v1.0.3) was applied to integrate samples across different conditions for further unsupervised clustering. For dimensionality reduction and clustering, the ‘FindNeighbors’ function was carried out with twenty principal components, and the ‘FindClusters’ function was performed with the resolution set to 0.3. Finally, uniform manifold approximation and projection (UMAP) was utilized to visualize the cell clusters, followed by the identification of differentially expressed genes for each cluster using the ‘FindAllMarkers’ function. To annotate the cell clusters, the DEGs of each cluster were combined with SingleR (v1.4.1) on the basis of the ImmGen database. The feature plot, dot plot and stacked bar plot of CD45^+^ immune cells were created with the ‘FeaturePlot’, ‘DotPlot’ and ‘ggplot’ functions, respectively.

For the reclustering analyses of the T-cell subpopulation, we extracted the CD4^+^ subsets from annotated CD45^+^ immune cells to systematically characterize the dynamics and diversity repertoire after MEKi treatment. Filtration, dimensionality reduction and unsupervised clustering were performed as described above. Following the identification of DEGs for each subcluster via the ‘FindAllMarkers’ function, the top discriminative marker genes were applied to annotate each subcluster. The UMAP plot was created by the ‘DimPlot’ function, and the UMAP density plot was plotted via the Nebulosa package (v1.0.1). To evaluate whether the indicated gene set was enriched in the expressed genes for each subcluster, the ‘AddModuleScore’ function was used to calculate the gene set activity score, and the ggpubr package (v0.6.0) was used for visualization. A volcano plot of the DEGs was visualized by scRNAtoolVis package (v0.0.7), and Heatmap was plotted with ‘DoHeatmap’ function. For the comparison between pre- and post-MEKi treatment, a stacked bar plot was generated to display the percentages of each subcluster via ggplot2 (v3.4.2), and a dot plot of the relative average expression of *Irf4* was generated via the ‘DotPlot’ function. Gene Ontology (GO) enrichment for DEGs was analyzed as described above and plotted via the ‘radarchart’ function of Fmsb (v0.7.5).

### Immune infiltration analysis

Published bulk RNA-seq data (GSE77940) of pre- and post-MKE inhibitor-treated (abbreviated as MKEi) tumor biopsies from four melanoma patients were used to perform immune infiltration analysis. First, differential expression analysis was conducted according to the previously described pipeline. The CIBERSORT algorithm [52] was subsequently utilized to estimate the infiltration ratios of immune cell subtypes via transcriptomic data from pre- and post-MKE inhibitor-treated tumor samples. Furthermore, the R package ggplot2 (v3.4.2) was used to visualize the immune cell infiltration scores.

### Human Tfh-related gene signatures

The characteristic gene sets associated with Tfh cells, B cells, and plasma cells as defined in previously published scRNA-seq atlases—namely, the immune cell atlas of human lung adenocarcinoma [53] and the pan-cancer tumor-infiltrating B cell atlas [36].

### TCGA data analyses

To evaluate survival outcomes, we obtained clinical metadata and transcriptomic data from the skin cutaneous melanoma (SKCM) cohort in The Cancer Genome Atlas (TCGA) via the TCGAbiolinks R package (v 2.23.2). The expression data were normalized with default parameters. Following initial data processing, marker genes of major cellular components of the humoral immune response were selected from the aforementioned scRNA-seq datasets to construct cell type-specific gene signatures, which were then employed to quantify immune infiltration scores in the TCGA-SKCM cohort by computing their mean expression levels. For cohort stratification, patients were divided into high- and low-infiltration groups at a 1:1 ratio based on their immune infiltration scores, with the median value serving as the cutoff. Finally, comparative survival analysis between the two groups was conducted using the survival (v3.2–10) and survminer (v0.4.9) R packages.

### Statistical analysis

All the statistical analyses were carried out with GraphPad Prism 9.2.0. Unless otherwise indicated, the data are presented as the means±SD. For comparisons between two groups, a two-tailed unpaired Student’s t test was used when the data fit a normal distribution and had similar variances. Welch’s t test was used for normally distributed data with different variances, and the Mann‒Whitney U test was used to test non-normally distributed data. For comparisons among multiple groups, p values were calculated via one-way or two-way ANOVA for normally distributed data and the Kruskal‒Wallis test for non-normally distributed data, followed by Dunnett’s or Sidak’s multiple comparisons test. Statistical significance is denoted in the figure legends by ns, not significant; *p ≤ 0.05; **p ≤ 0.01; ***p ≤ 0.001; ****p ≤ 0.0001.

## Results

### The MEK1/2 signaling pathway is differentially activated in Tfh and non-Tfh cells

**Fig. 1:**
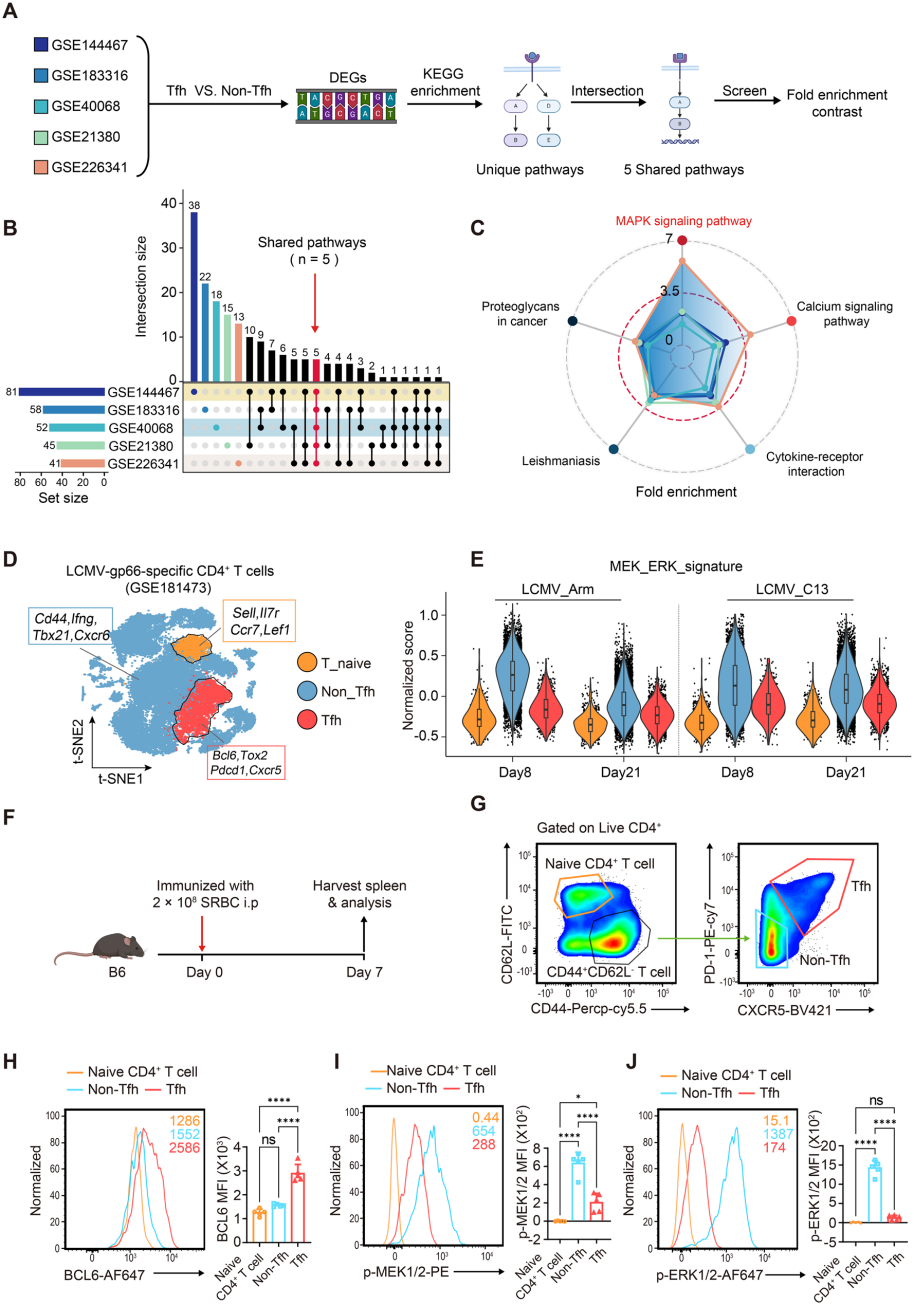
The MEK1/2 signaling pathway is differentially activated in Tfh and non-Tfh cells. (**A**) Overview of the analysis workflow. (**B**) UpSet plot comparing multiple differentially enriched pathways, including the total number of enriched pathways in different public GSE datasets (colored bars in the bottom left corner); the histogram above each plot indicates the number of enriched pathway terms in each category, and pathways shared among ≥ 5 public GSE datasets are highlighted with red dots. (**C**) Radar plot showing the Fold enrichment of 5 shared pathways obtained from the intersection in (B). Fold enrichment represents the degree of enrichment of KEGG terms in different public GSE datasets, and the MAPK signaling pathway (highlighted in red font) was identified as the most enriched term across 5 shared pathways. (**D**) TSNE plot showing the 3 subclusters generated from murine LCMV-gp66-specific CD4^+^ T cells from the published scRNA-seq dataset GSE181473; representative signature genes of each cluster are highlighted. (**E**) Violin plot showing the MEK_ERK feature score of each cluster at the indicated time points under two different conditions. (**F**) Experimental scheme of the SRBC immunization model. (**G**) Gating strategy for the analysis of CD62L^+^CD44^-^ naïve CD4^+^ T cells and PD-1^+^CXCR5^+^ Tfh and PD-1^-^CXCR5^-^ non-Tfh subsets in the spleens of SRBC-immunized mice. (**H**) Representative histograms of BCL6 expression in naïve CD4^+^, Tfh and non-Tfh cells. The mean fluorescence intensity (MFI) is summarized on the right (*n* = 5). (**I**-**J**) Representative histograms of p-MEK1/2 and p-ERK1/2 in naïve CD4^+^, Tfh and non-Tfh cells are shown (*n* = 5). The data are representative of at least three independent experiments. The error bars represent the means ± SD; *p* values in (H-J) were calculated using one-way ANOVA followed by Tukey’s multiple-comparisons test; *, *p* < 0.05; **, *p* < 0.01; ***, *p* < 0.001; ****, *p* < 0.0001; ns, no significance

Given that various signaling pathways have been proposed as essential regulators of the development and function of Tfh cells [54], we sought to investigate the underlying mechanisms by which signaling pathways are involved in the regulation of Tfh differentiation. First, we searched 5 published murine RNA sequencing (RNA-Seq) datasets derived from various representative immune models, including lymphocytic choriomeningitis virus (LCMV) viral infection, KLH/CFA immunization and adoptive Armstrong infection experiments [55–59]. Differentially expressed genes (DEGs) were identified by an expression change of at least 1.5-fold and an adjusted *p* value of 0.05 in Tfh versus non-Tfh cells, followed by KEGG pathway enrichment analysis. To determine which pathways exhibited common patterns, we compared multiple differentially enriched pathways among the 5 datasets. More specifically, pathways shared among ≥ 5 published GSE datasets were selected and evaluated by calculating the respective fold enrichment values (Fig. 1A). Similar to previous studies, we identified 5 shared pathways, including cytokine‒cytokine receptor interactions, proteoglycans in cancer, leishmaniasis, the calcium signaling pathway, and the MAPK signaling pathway (Fig. 1B). Notably, the MAPK signaling pathway (mmu04010) was identified as the most enriched pathway across the 5 shared pathways (Fig. 1C). Consistent with the results obtained by evaluating murine transcriptional profiles, we also found that the term regulation of MAPK activity (GO:0043405) was enriched by GO enrichment analysis of the DEGs upregulated in Tfh cells, as determined on the basis of the RNA-seq profiles of human tonsil primary Tfh cell and non-Tfh cells derived from the published dataset GSE58596 (Fig. S1, A and B) [60]. To further confirm the specific role of MAPK signaling activation in the T-cell-dependent humoral response, we utilized an unbiased scRNA-seq analysis of 39,056 LCMV-gp66-antigen-specific CD4^+^ T cells upon infection with either LCMV-C13 or Armstrong (GSE181473) [3]. After dimensionality reduction and clustering, three major subpopulations were annotated based on feature genes (Fig. S1, C and D). Among these, the naïve CD4^+^ T-cell subpopulation presented high expression of resting state-related genes such as *Sell*, *Il7r*, *Ccr7*, and *Lef1*, whereas the activated *Cd44*^*high*^ CD4^+^ T-cell cluster was further categorized into Tfh and non-Tfh subpopulations (Fig. 1D). Pathway activity analysis revealed that MEK-ERK signature scores were significantly lower in the Tfh subset than in non-Tfh cells, indicating differential activation of this pathway between the two subsets (Fig. 1E). Collectively, the results of these bioinformatics analyses suggest that the MEK-ERK pathway is critical for Tfh cell development and function.

To validate these findings of the above bioinformatics analysis, we next immunized C57BL/6 mice with sheep red blood cells (SRBCs) via i.p. injection, which can induce robust polyclonal GC responses (Fig. 1, F and G). After 7 days of SRBC immunization, we detected significantly increased expression of BCL6 among CD4^+^CD62L^-^CD44^+^PD-1^+^CXCR5^+^ Tfh cells in the spleen via flow cytometry analysis (FCM) (Fig. S1E). Next, we investigated the levels of activated phosphorylated MEK1/2 and phosphorylated ERK1/2 among CD62L^+^CD44^-^ naïve CD4^+^ T cells and PD-1^-^CXCR5^-^non-Tfh and PD-1^+^CXCR5^+^ Tfh subpopulations in the spleens of SRBC-immunized mice. Unexpectedly, the Tfh cell population exhibited a lower mean fluorescence intensity (MFI) of phosphorylated MEK1/2 and phosphorylated ERK1/2 than non-Tfh cells did (Fig. 1, H-J). Previous studies have demonstrated that ERK1/2 is a potent negative modulator of Tfh cell differentiation [61]. Considering these findings in combination with our study results, we speculated that MEK1/2 signaling may also play an essential role in Tfh cell fate programming.

### Codeletion of MEK1 and MEK2 promotes the differentiation of Tfh cells in vitro

**Fig. 2:**
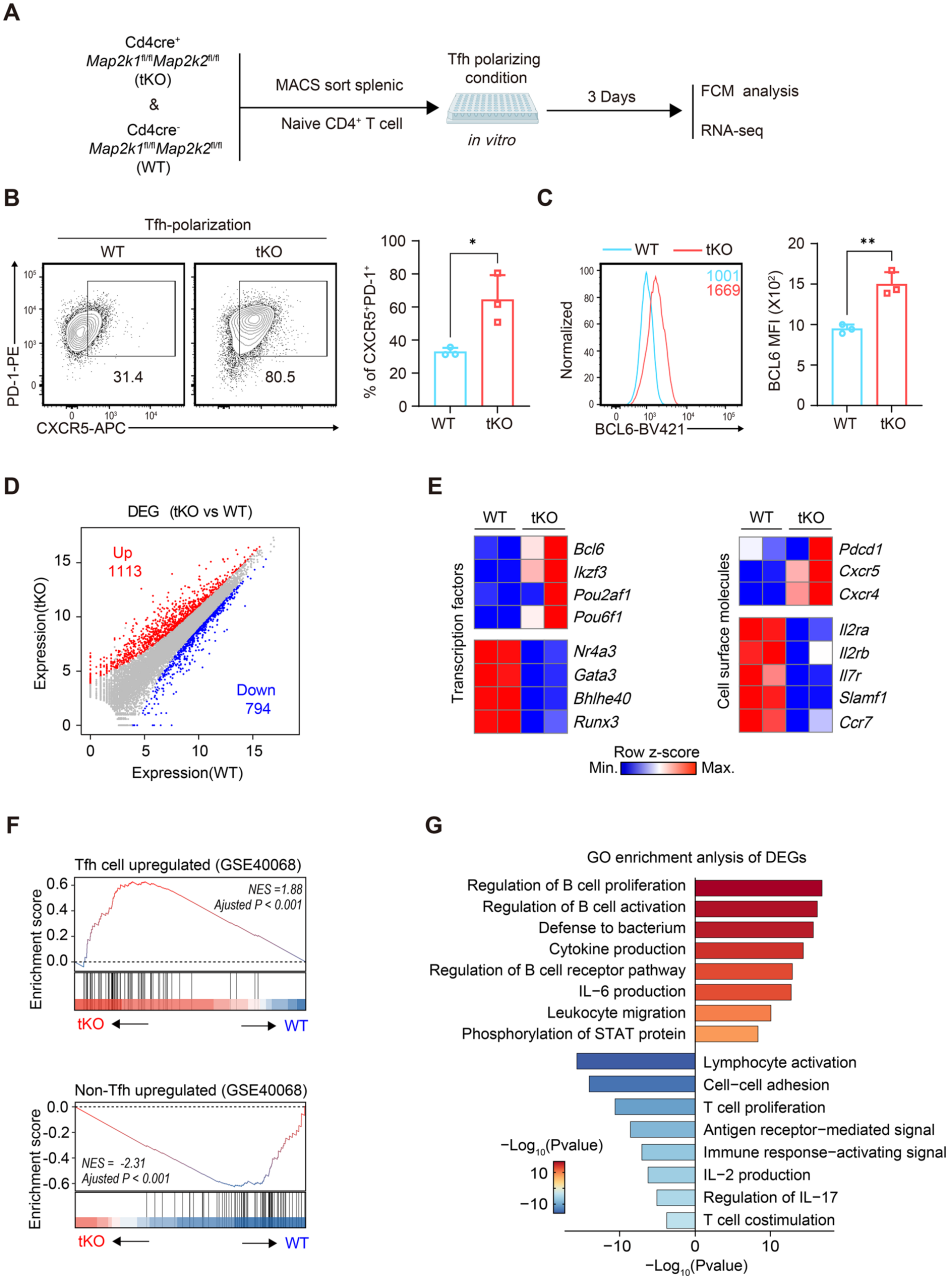
Codeletion of MEK1 and MEK2 promotes Tfh differentiation in vitro. (**A**) Schematic of in vitro Tfh polarization. (**B**) Representative contour plots and bar plots of the percentages of Tfh (CXCR5^+^PD-1^+^ cells) cells from WT or *Mek*1/2-tKO mice on day 3 post-polarization (*n* = 3). (**C**) Representative histograms and bar plots of BCL6 expression in CXCR5^+^PD-1^+^ cells from WT or *Mek*1/2-tKO mice on day 3 post-polarization (*n* = 3). (**D**) Scatter plots of genes whose expression was upregulated (red) or downregulated (blue) by 1.5-fold or more in the *Mek*1/2-tKO group than in the WT group under Tfh-skewing culture conditions, as determined by RNA-seq analysis. (**E**) Heatmap showing the relative expression of selected genes of interest in *Mek*1/2-tKO versus WT Tfh-polarized cells. (**F**) Gene set enrichment analysis of the transcriptional profiles of *Mek*1/2-tKO versus WT Tfh-polarized cells on the basis of the Tfh-upregulated signature gene set (top) and the non-Tfh-upregulated signature gene set (bottom), as previously described by Liu et al. (GSE40068). (**G**) Gene Ontology enrichment analysis of DEGs in *Mek*1/2-tKO versus WT Tfh-polarized cells. The data are representative of three independent experiments. The error bars represent the means ± SD; *p* values in (B-C) were calculated using unpaired two-tailed t test; *, *p* < 0.05; **, *p* < 0.01; ***, *p* < 0.001; ****, *p* < 0.0001; ns, no significance

Considering the redundant roles of MEK1 and MEK2 [62], we generated T-cell-specific *Mek*1/2-deficient mice by crossing *Cd4-Cre*^+^ mice with *Map2k1* and *Map2k2* floxed mice, eventually obtaining *Cd4-Cre*^+^*Map2k1*^fl/fl^*Map2k2*^fl/fl^ double-gene knockout mice (abbreviated as *Mek*1/2-tKO mice) to better dissect the role of MEK1/2 signaling in Tfh cell differentiation (Fig. S2A). *Mek*1/2-tKO mice were developmentally normal and fertile, and their genotypes were subsequently determined via PCR of tail DNA (Fig. S2B). P-MEK1/2 or p-ERK1/2 expression among naïve splenic CD4^+^ T cells was confirmed by FCM (Fig. S2, C-D). Given that a previous study demonstrated that MAP-kinase pathway signaling may influence T-cell lineage development in the thymus [63], we first aimed to evaluate T-cell development in *Mek*1/2-tKO and littermate control WT mice. Indeed, the percentage of CD4 single-positive (CD4^+^CD8a^-^) cells in thymocytes (Fig. S2E) and the percentage of CD4^+^TCR-β^+^ cells in splenocytes (Fig. 2F) were partially lower in Mek1/2-tKO mice than in WT mice, indicating that *Mek*1/2 is dispensable for the development of T cells (Fig. S2E). Overall, despite some limitations, these data indicate that *Mek*1/2-tKO mice are suitable for further investigation of Tfh cell differentiation.

Next, we analyzed the function of MEK1/2 signaling in Tfh cell development more specifically via in vitro Tfh polarization experiments. First, naïve CD4^+^ T cells were sorted from the spleens of WT and *Mek*1/2-tKO mice and then cultured under anti-CD3 and anti-CD28 stimulation coupled with Tfh cell skewing conditions to induce Tfh differentiation (Fig. 2A). After 3 days, compared with the WT control group, the *Mek*1/2-tKO group presented an obvious increase in the percentage of Tfhs (identified as CXCR5^+^PD-1^+^ cells) (Fig. 2B). Moreover, we detected significantly increased BCL6 expression in polarized *Mek*1/2-tKO CD4^+^ T cells compared with that in their WT counterparts (Fig. 2C). Overall, our in vitro findings suggested that T-cell-specific *Mek*1/2 deletion promotes Tfh differentiation.

In addition, we performed RNA-Seq analysis of in vitro cultured WT or *Mek*1/2-tKO CD4^+^ T cells on day 3 post-polarization. Compared with that in polarized WT CD4^+^ T cells, the expression of 1113 genes were upregulated, and that of 794 genes was downregulated in polarized *Mek*1/2-tKO CD4+ T cells (Fig. 2D). Notably, representative characteristic Tfh cell transcription factors (*Bcl6*, *Ikzf3*) and cell surface molecules (*Pdcd1*, *Cxcr5*) were upregulated in the *Mek*1/2-tKO group, whereas Non-Tfh cell-characteristic transcription factors (*Gata3*, *Bhlhe40*) and cell surface molecules (*Il2ra*, *Il7r*) were downregulated (Fig. 2E). We subsequently performed gene set enrichment analysis (GSEA) and revealed that genes in the “Tfh-upregulated signature gene set” were enriched in the transcriptome of the *Mek*1/2-tKO group, whereas the “non-Tfh-upregulated signature gene set”, as previously described by Liu et al., exhibited the reverse enrichment pattern (Fig. 2F) [56]. Additionally, global evaluation via Gene Ontology (GO) enrichment analysis of the DEGs illustrated the immunological programs of Tfh cells in the absence of MEK1/2 signaling. Among these pathways, the top enriched pathways associated with the upregulated genes included the regulation of B-cell activation, proliferation, and the B-cell receptor pathway (Fig. 2G). In conclusion, our results suggest that the codeletion of MEK1 and MEK2 promotes Tfh differentiation, which is accompanied by alterations in Tfh transcriptional profiles in vitro.

Notably, RNA-seq data of *Mek*1/2-tKO CD4^+^ T cells cultured under Tfh polarizing conditions revealed not only augmented Tfh cell programming, but also reduced the expression of key genes in other CD4 subsets (Fig. 2E). Given that MEK/ERK signaling is central upstream of the differentiation of a broad range of CD4^+^ T cell subsets, we decided to determine whether this was a genuine fate of the lineage or a secondary consequence of weakened signaling. First, naïve CD4^+^ T cells were sorted from the spleens of WT and *Mek*1/2-tKO mice and then cultured under anti-CD3 and anti-CD28 stimulation coupled with various cell skewing conditions to induce different Th differentiation (Fig. S2I). After 3 days, compared with the WT control group, the *Mek*1/2-tKO group presented an obvious decrease in the percentage of IFN-γ^+^ cells and IL-17A^+^ cells among live CD4^+^ cells. On the contrary, we detected significantly increased Foxp3+ cells among live CD4^+^ cells in polarized *Mek*1/2-tKO group (Fig. S2J-K). Overall, our in vitro findings suggested that *Mek*1/2-tKO T cells showed an impaired ability to differentiate into Th1 and Th17 cells, whereas they promoted Treg generation. Frequencies of key cytokine-producing cells of all major CD4 Th subsets were comparable suggesting MEK1/2 to be dispensable for regulating differentiation of those Th subsets in vitro. Considering the spatiotemporal dynamics and plasticity of T cell differentiation, along with the typically mutually exclusive developmental paths of Tfh and Th1 cells [64], we hypothesized that the promotion of Tfh cell differentiation by *Mek*1/2 ablation may involved both the active enforcement of the Tfh program and the passive redistribution of lineage potential resulting from impaired alternative CD4^+^ T cell differentiation.

### Codeletion of MEK1 and MEK2 promotes Tfh differentiation in vivo

**Fig. 3:**
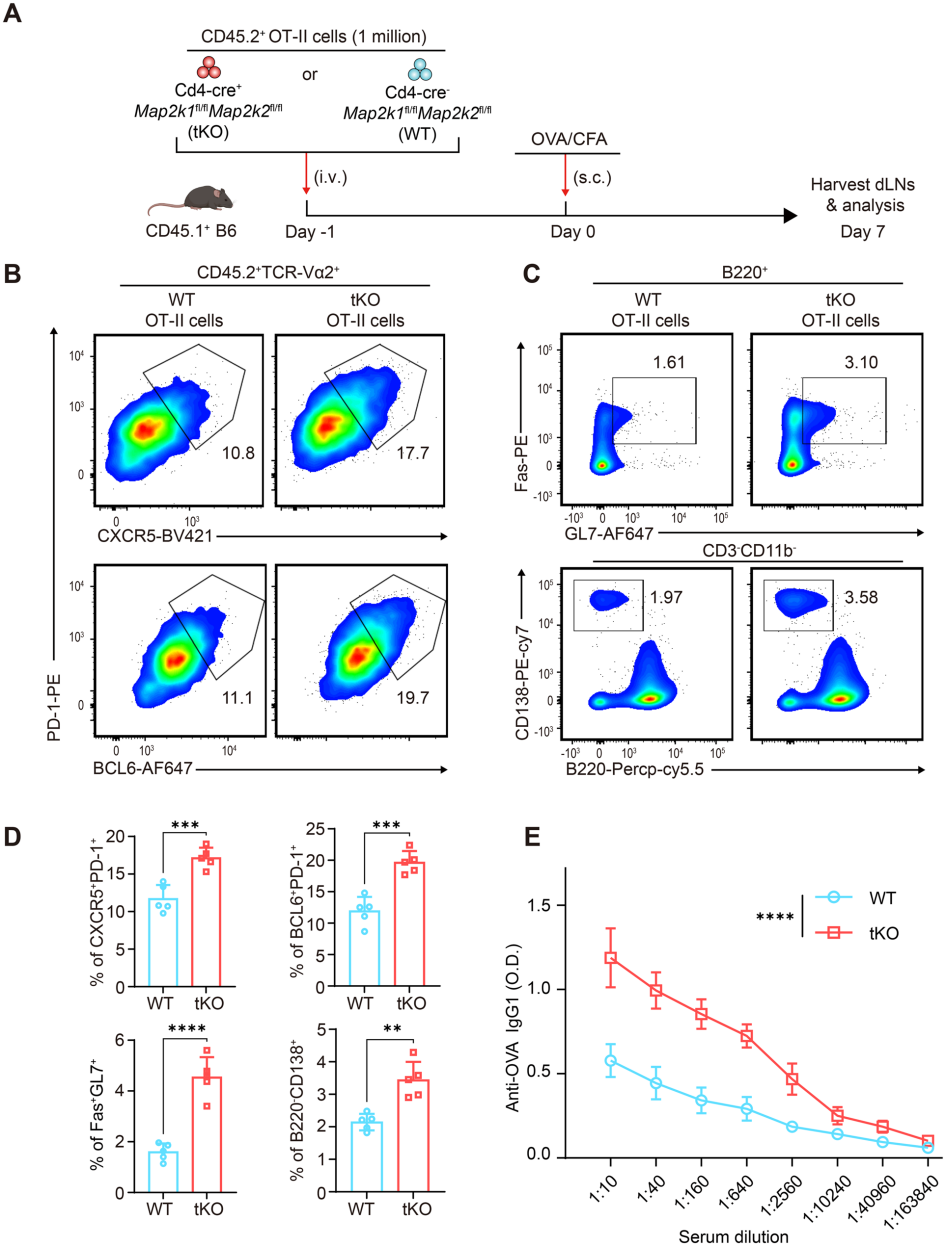
Codeletion of MEK1 and MEK2 potentiates Tfh differentiation in vivo. (**A**) Schematic of in vivo OVA/CFA immunization experiments. (**B**) Representative psuedocolor plots showing the percentages of CXCR5^+^PD-1^+^ and BCL6^+^PD-1^+^ cells among CD45.2^+^TCR-Va2^+^ cells from recipient mice in the dLns on day 7 after OVA immunization (*n* = 5). (**C**) Representative psuedocolor plots showing the percentages of Fas^+^GL7^+^ cells among B220^+^ cells and B220^-^CD138^+^ cells among CD3^-^CD11b^-^ cells from recipient mice in the dLns on day 7 after OVA immunization (*n* = 5). (**D**) Representative bar plots showing the percentages of CXCR5^+^PD-1^+^ (Tfh), BCL6^+^PD-1^+^ (Tfh), Fas^+^GL7^+^ (GC B), and B220^-^CD138^+^ (plasma) cells. (**E**) Detection of the serum levels of anti-OVA-specific IgG1 by ELISA after 7 days of OVA immunization (*n* = 5). The data are representative of at least three independent experiments. The error bars represent the means ± SD; *p* values in (D) were calculated using unpaired two-tailed t test; *p* value in (E) was calculated using two-way ANOVA with šídák’s multiple comparison; *, *p* < 0.05; **, *p* < 0.01; ***, *p* < 0.001; ****, *p* < 0.0001; ns, not significant

To further explore the T-cell-specific role of *Mek*1/2 in regulating Tfh cell fate programming and germinal center (GC) B responses, we generated *Mek*1/2-tKO OT-II mice by crossing the *Mek*1/2-tKO strain with the OT-II transgenic strain (OVA-specific TCR). Considering that T-cell-specific *Mek*1/2 ablation can influence T-cell lineage development in the thymus, we employed an adoptive transfer strategy (Fig. 3A). Briefly, 1 million CD45.2^+^ WT or *Mek*1/2-tKO OT-II T cells were adoptively transferred into CD45.1^+^ B6 recipient mice one day ahead, after which the recipient mice were subjected to OVA/CFA immunization. On day 7 after immunization, we assessed Tfh cell differentiation in the draining lymph nodes (dLNs) of the recipient mice. Genetic ablation of *Mek*1/2 in OT-II cells did not affect their proportion during adoptive transfer experiments (Fig. S2, G-H). In line with the findings obtained from the Tfh polarization experiment in vitro, *Mek*1/2-tKO OT-II T cells showed significantly elevated percentages of CXCR5^+^PD-1^+^ and BCL6^+^PD-1^+^cells among CD45.2^+^TCR-Vα2^+^ cells after OVA immunization in vivo (Fig. 3B). In addition, the percentages of Fas^+^GL7^+^ cells among B220^+^ cells and B220^-^CD138^+^ cells among CD3^-^CD11b^-^ cells also increased as a result of *Mek*1/2 ablation in OT-II T cells (Fig. 3C). Consistent with the increased GC response, OVA-specific IgG1 production in the serum of OVA-immunized recipient mice was also markedly elevated (Fig. 3E). Taken together, our data revealed that codeletion of MEK1 and MEK2 potentiates Tfh differentiation and GC B responses in a T-cell-intrinsic manner.

### Promotion of Tfh cell differentiation by *Mek*1/2 deletion depends on the epigenetic regulation of IRF4

**Fig. 4:**
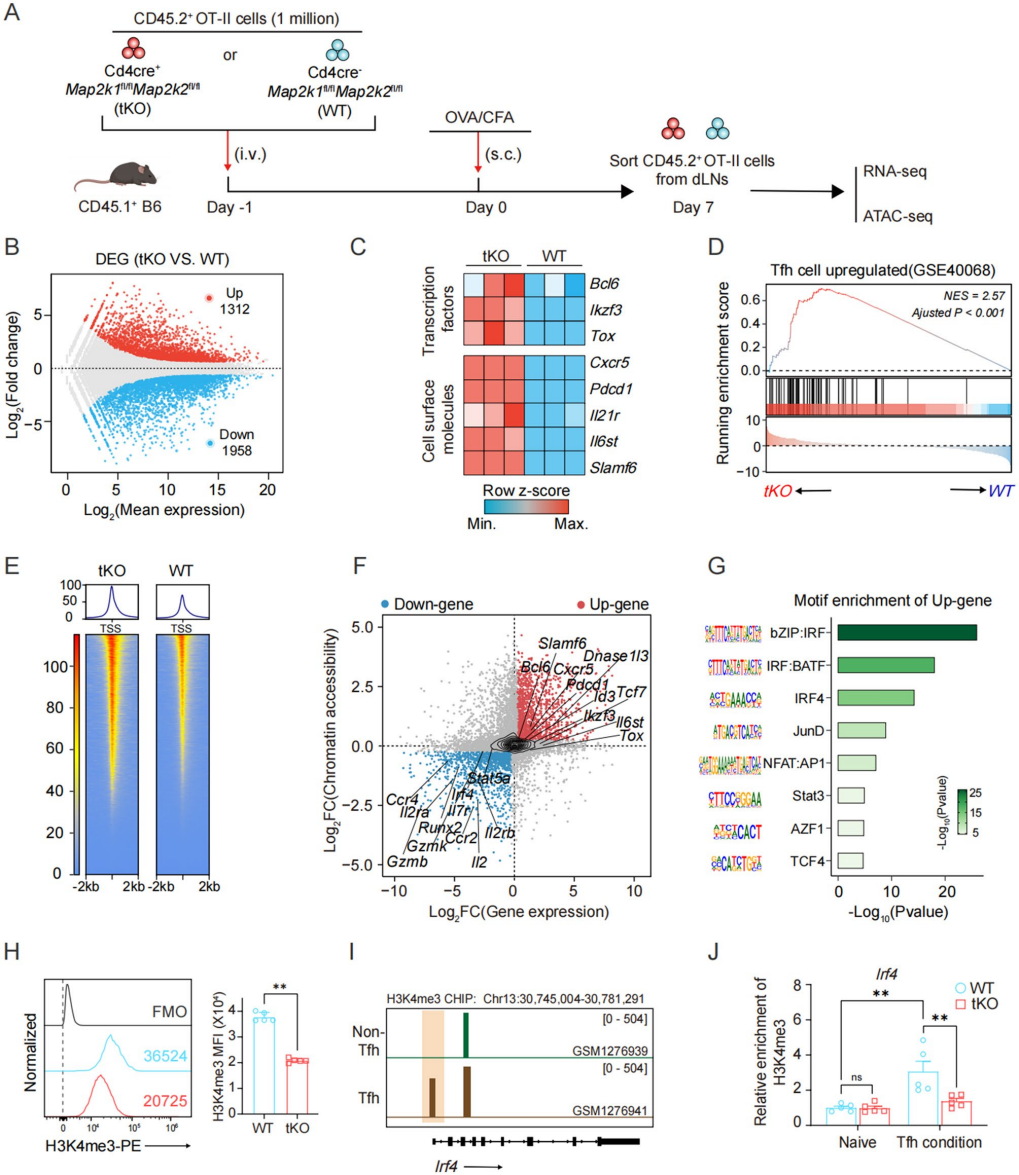
Integrative RNA-seq and ATAC-seq analyses identified IRF4 as a modulator of the promotion of Tfh cell differentiation by *Mek*1/2 deletion. (**A**) Schematic of sequencing sample preparation. (**B**) MA plots displaying differentially expressed genes in transferred *Mek*1/2-tKO versus WT CD45.2^+^OT-II T cells characterized by RNA-seq; upregulated (red dots) and downregulated (blue dots) DEGs are highlighted. (**C**) Heatmap displaying the relative expression of Tfh cell characteristic genes in transferred *Mek*1/2-tKO versus WT CD45.2^+^OT-II T cells. (**D**) Gene set enrichment analysis of the transcriptional profiles of transferred *Mek*1/2-tKO versus WT CD45.2^+^OT-II T cells on the basis of the Tfh-upregulated gene signature (top), as previously described by Liu et al. (GSE40068). (**E**) Heatmap displaying ATAC-seq read densities within ±2 kb of the peak center for differentially accessible chromatin regions from transferred *Mek*1/2-tKO versus WT CD45.2^+^OT-II T cells. The histograms above represent the average read density across the same intervals for each sample group. (**F**) Scatterplots displaying the correlation of the peak accessibility of the nearest genes versus the differential expression of the genes. Briefly, peak log_2_(FC) scores from ATAC-seq Deseq2 analysis and gene log_2_(FC) scores from RNA-seq Deseq2 analysis were tabulated and gene-matched. (**G**) HOMER motif enrichment analysis of the DNA sequences flanking the up gene identified from (F). Consensus islet-related motifs (motif), transcription factor names (name), and *p* values are shown. (**H**) Representative FCM histograms (left panel) of H3K4me3 MFI on transferred CD45.2^+^ WT or Mek1/2-tKO OT-II T cells in dLns from recipient mice on day 7 post-immunization, MFI were depicted alongside (*n* = 5). (**I**) Integrative genome viewer (IGV) displaying genome-wide histone modifications (H3K4me3,permissive marker) at the *Irf4* locus in non-Tfh and Tfh cells from draining LNs of keyhole limpet hemocyanin (KLH) immunized mice (H3K4me3 ChIP-Seq: GSE52840). (**J**) ChIP-qPCR analysis of the relative enrichment of H3K4me3 on the *Irf4* promotor in splenic CD4^+^ T from WT or *Mek*1/2-tKO mice on day 3 post-Tfh polarization (*n* = 3). The data are representative of at least three independent experiments. The error bars represent the means ± SD; *p* values in (H) were calculated using mann-whitney u test; *p* value in (J) was calculated using two-way ANOVA with šídák’s multiple comparison; *, *p* < 0.05; **, *p* < 0.01; ***, *p* < 0.001; ****, *p* < 0.0001; ns, not significant

To further elucidate the molecular mechanisms underlying *Mek*1/2 deficiency-mediated Tfh differentiation, we performed RNA-seq combined with ATAC-seq analysis of transferred CD45.2^+^ WT or *Mek*1/2-tKO OT-II T cells in dLNs from recipient mice on day 7 postimmunization (Fig. 4A). First, principal component analysis (PCA) was applied to confirm that the transferred CD45.2^+^*Mek*1/2-tKO OT-II T cells substantially differed from those in the WT control group in terms of the transcriptional profile (Fig. S3A). Moreover, compared to the WT control group, 1312 genes were upregulated, and 1958 genes were downregulated in the transferred CD45.2^+^*Mek*1/2-tKO OT-II T cells (Fig. 4B). Consistent with the results obtained from RNA-seq in vitro, representative Tfh cell-characteristic transcription factors (*Bcl6*, *Ikzf3, Tox*) and cell surface molecules (*Pdcd1*, *Cxcr5, Il21r, Il6st*) were also upregulated in the transferred *Mek*1/2-tKO OT-II group in vivo (Fig. 4C). Subsequently, GSEA of the DEGs revealed enrichment of the “Tfh-upregulated signature gene set” in the transcriptome of the transferred *Mek*1/2-tKO OT-II group (Fig. 4D). Additionally, global evaluation via GO enrichment analysis of the DEGs of the upregulated and downregulated genes of transferred *Mek*1/2-tKO CD45.2^+^OT-II T cells. Among these pathways, upregulated genes are primarily enriched in pathways related to Tfh and humoral immunity, whereas downregulated genes are associated with pathways governing effector CD4^+^ T cell phenotypes, such as Th1 and Th17 lineages (Fig. S3G-H).

Furthermore, we explored whether the observed changes in the expression of genes related to Tfh cells at the transcriptional level were correlated with alterations in the accessibility of the corresponding chromatin. First, Pearson’s correlation analyses based on differentially accessible chromatin regions revealed obvious distinctions across different ATAC-seq samples (Fig. S3B). Compared with the WT control group, we observed 27,670 differentially accessible chromatin regions across the transferred CD45.2^+^ OT-II T cells, which were distributed mainly in the gene body (40.7%), intergenic regions (28.2%), or promoter regions (31.1%) (Fig. S3D). Moreover, the ATAC signal within the ±2 kb region flanking the TSS was generally more accessible in the *Mek*1/2-tKO OT-II group than in the WT control group (Fig. 4E). Notably, the expression of Tfh cell-characteristic genes, such as *Icos*, *Il6st*, *Il21* and *Tox2*, clearly increased in the *Mek*1/2-tKO OT-II group (Fig. S3F). Overall, the increase in chromatin accessibility around the Tfh lineage-specific gene locus was also accompanied by increases in the expression of relevant genes, indicating that there are potential epigenetic regulatory mechanisms involved in T-cell-specific *Mek*1/2 ablation-mediated Tfh cell fate programming.

After the transcriptional changes and chromatin accessibility profiles were systematically described, we next investigated the possible regulators driving Tfh differentiation under T-cell-specific *Mek*1/2 ablation conditions. First, combined analysis of RNA-seq and ATAC-seq data based on Pearson’s correlation coefficient confirmed the positive correlation of transcriptional and epigenetic changes (Pearson *r* = 0.26, p value < 2.2 × 10^−16^) (Fig. S3C). To link *Mek*1/2 deficiency-associated chromatin changes to transcriptional levels, ATAC-seq profiles of transferred CD45.2^+^*Mek*1/2-tKO or WT OT-II T cells were generated to match their RNA-seq counterparts. Briefly, peak log_2_FoldChange scores from ATAC-seq Deseq2 analysis and gene log_2_FoldChange scores from RNA-seq Deseq2 analysis were tabulated and gene-matched (Fig. 4F). In total, 1678 genes presented upregulation, and 1107 genes presented downregulation in a manner consistent with simultaneous transcriptional and epigenetic changes; these genes are hereafter referred to as the “UP-gene” and “Down-gene” subcategories, respectively.Furthermore, we performed HOMER motif enrichment analysis of the DNA sequences flanking the identified “UP gene” set based on known motif datasets. Notably, binding sites for multiple members of the IRF family, such as bZIP: IRF, IRF:BATF and NFAT:AP1, were obviously enriched in these regions (Fig. 4G). As numerous previous studies have reported that IRF4 plays pivotal roles in various aspects of T-cell differentiation and function [65], we focused our investigation on the candidate transcription factor IRF4.

Based on the results of the omics screening, we next examined changes in IRF4 expression following *Mek*1/2 deletion. First, we compared normalized *Irf4* expression at the transcriptional level and contrasted the corresponding *Irf4* chromatin accessibility at the epigenomic level. As expected, compared with their WT OT-II counterparts, transferred CD45.2^+^*Mek*1/2-tKO OT-II T cells presented obviously lower *Irf4* chromatin accessibility peaks coupled with decreased transcription (Fig. S6D). Moreover, we detected a lower IRF4 MFI in the transferred *Mek*1/2-tKO OT-II T cells than in the WT OT-II T cells in the dLNs of the recipient mice on day 7 post-immunization (Fig. S6E). Overall, these data indicate that *Irf4* underwent epigenetic, transcriptional and protein decreases in T-cell-specific *Mek*1/2 ablation conditions.

Next, we further explored the potential mechanism by which MEK1/2 regulates IRF4 during Tfh cell development.Our existing omics data indicates that MEK1/2 deletion leads to reduced *Irf4* expression and altered chromatin accessibility. Consistent with our observations, Esnault et al. demonstrated that in mouse embryonic fibroblasts, ERK-induced histone modifications at transcription start sites (TSSs) initiate a chromatin modification cascade associated with transcription [66]. To further elucidate this epigenetic regulation, we detected the levels of four common active histone modifications of transferred CD45.2^+^ WT or *Mek*1/2-tKO OT-II T cells in dLNs from recipient mice on day 7 post-immunization. Although no statistical differences in H3K36me3, H3K9ac and H3K27ac levels were observed (Fig. S6A-C), the level of H3K4me3 was markedly decreased in *Mek*1/2-tKO OT-II T cells (Fig. 4H). A wealth of evidence has established that H3K4me3, a typical marker of transcriptionally active chromatin [67], plays a key role in determining T cell lineage fates [68]. To clarify whether *Irf4* is regulated by H3K4me3 modification, we analyzed the epigenetic features of the *Irf4* promotor locus and found that it was marked with active chromatin marker H3K4me3 in Tfh cells from draining LNs of keyhole limpet hemocyanin (KLH) immunized mice (H3K4me3 ChIP-Seq: GSE52840) (Fig. 4I). In addition, to directly clarify that MEK1/2 deletion decreases IRF4 expression by reducing the enrichment of H3K4me3 at the *Irf4* promotor, a ChIP assay was conducted using the H3K4me3 antibody. We found that the enrichment of H3K4me3 at the *Irf4* promotor was significantly attenuated in *Mek*1/2-tKO Tfh cells under in vitro Tfh polarized condition (Fig. 4J).

Overall, these results indicated that MEK1/2 signaling influences the epigenetic state at the *Irf4* promoter locus during Tfh development, likely through indirect mechanisms downstream of the signaling cascade, thereby diminishing IRF4 chromatin accessibility and transcription. This MEK1/2-IRF4 axis may further contributing to Tfh development upon OVA/CFA immunization.

### *Irf4* overexpression counteracts the promotion of Tfh cell differentiation by *Mek*1/2 ablation

**Fig. 5:**
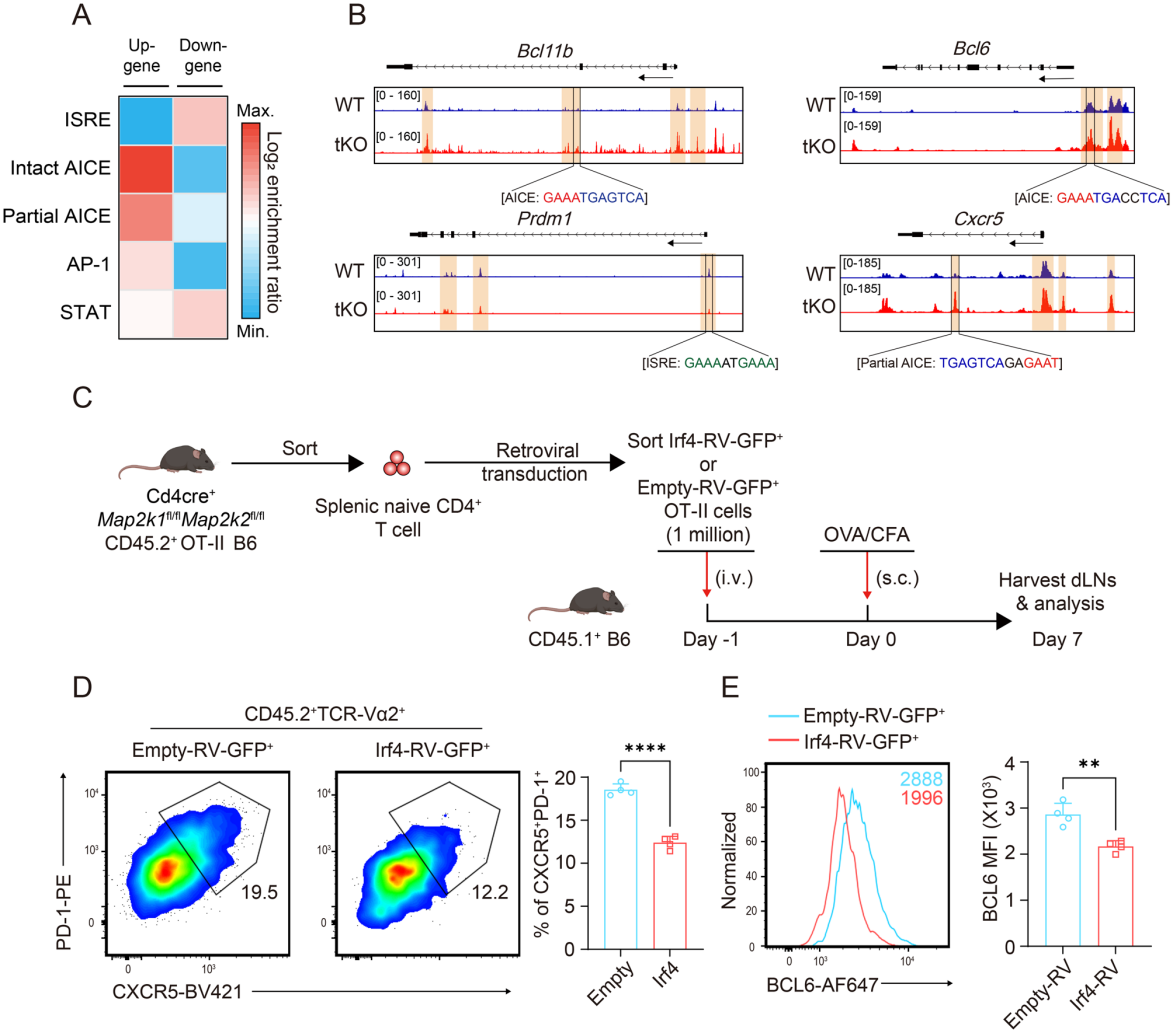
Overexpressing *Irf4* counteracts the promotion of Tfh cell differentiation by *Mek*1/2 ablation. (**A**) Heatmap displaying the enrichment ratio (Log_2_) of given Irf4 DNA binding motifs (y-axis) in the DNA sequences flanking the up- and downgenes (x-axis) identified from ATAC-seq and RNA-seq. (**B**) IGV displaying representative ATAC-seq signal tracks in the Tfh and non-Tfh cell-characteristic gene loci from transferred *Mek*1/2-tKO versus WT CD45.2^+^OT-II T cells. Representative AICE- or ISRE-binding motifs are highlighted below the corresponding differentially accessible peaks. (**C**) Schematic of the *Irf4* overexpression experimental design. Sorted splenic CD45.2^+^*Mek*1/2-tKO OT-II^+^ naïve CD4^+^ T cells were retrovirally transduced with empty or RV-*Irf4-*GFP^+^ vectors. One million infected cells were transferred into CD45.1^+^ B6 mice on day 1, and then, the recipient mice were subjected to OVA/CFA immunization on day 0, followed by FCM analyses of CD45.2^+^ OT-II T cells in the dLns on day 7. (**D**) representative psuedocolor plots displaying the percentages of CXCR5^+^PD-1+ cells among empty or RV-*Irf4*-GFP^+^ vectors retrovirally transduced with CD45.2^+^TCR-Va2^+^ cells in the dLns on day 7 after OVA immunization (*n* = 4). (**E**) representative histograms and bar plots of BCL6 expression in CXCR5^+^PD-1^+^ cells from empty or RV-*Irf4*-GFP^+^ vectors retrovirally transduced with CD45.2^+^TCR-Va2^+^ cells in the dLns on day 7 after OVA immunization (*n* = 4). The data are representative of at least three independent experiments. The error bars represent the means ± SD; *p* values in (D-E) were calculated using an unpaired two-tailed t test; *, *p* < 0.05; **, *p* < 0.01; ***, *p* < 0.001; ****, *p* < 0.0001; ns, not significant

Given that Krishnamoorthy et al. reported that IRF4 targets divergent DNA motifs in a dose-dependent manner to control Tfh and Teff cell gene programs [14], we also adopted their method to evaluate IRF4 target genes and binding sites in the absence of *Mek*1/2. Specifically, we first searched for IRF4 binding sequence motifs in DNA sequences flanking the identified “UP-gene” and “Down-gene” subcategories via the use of position weight matrices representing the AP-1, STAT, and ISRE elements and two versions of the AICE element. By calculating the proportion of chromatin-accessible regions (ChARs) in a subcategory that contained a motif versus the same proportion in all ChARs, we finally obtained the subcategory-specific enrichment of each motif (Fig. 5A). Partial and intact AICE motifs were enriched mainly in the “UP-gene” subcategory, whereas the ISRE motif was highly enriched in the “Down-gene” subcategory. To deepend our molecular analysis, we compared the chromatin accessibility of Tfh and non-Tfh cell-characteristic genes at potential AICE or ISRE loci via an integrative genome viewer (Fig. 5B). Notably, key genes, such as *Bcl6, Cxcr5* and *Bcl11b,* exhibited obviously greater chromatin accessibility peaks at the potential partial and intact AICE loci in the *Mek*1/2-tKO OT-II group. In contrast, the non-Tfh key gene *Prdm1* showed increased chromatin accessibility peaks at potential ISRE loci in the WT OT-II group. Collectively, these omics analyses implied that IRF4-dependent epigenetic modulation of Tfh cell-characteristic genes might serve as the driving force triggering Tfh transcriptional reprogramming, which is responsible for T-cell-specific *Mek*1/2 ablation-mediated Tfh differentiation. Specifically, low expression of IRF4 may lead to binding to potential high-affinity recognition elements (such as AICE) at Tfh cell-characteristic gene loci and increase their transcription.

To validate whether *Irf4* epigenetic modulation is the predominant mechanism by which *Mek*1/2 deficiency mediates Tfh cell fate programming, we firstly confirmed the function of *Irf4* via overexpression experiments. Specifically, we first sorted splenic naïve CD4^+^ T cells from CD45.2^+^*Mek*1/2-tKO OT-II^+^ B6 mice and induced the expression of *Irf4* via retroviral transduction with empty or RV-*Irf4*-GFP^+^ vectors. Subsequently, 1 million infected cells were transferred to CD45.1^+^B6 mice on day 1, and then, the recipient mice were subjected to OVA/CFA immunization on day 0, followed by FCM analyses of CD45.2^+^ OT-II T cells in the dLNs on day 7 (Fig. 5C). Notably, in the *Irf4* transduction group, CD45.2^+^*Mek*1/2-tKO OT-II^+^ T cells presented a significant decrease in the percentage of Tfhs (identified as CXCR5^+^PD-1^+^ cells), indicating that the promotion of Tfh cell differentiation by Mek1/2 ablation was counteracted by *Irf4* overexpression (Fig. 5D). Consistent with this result, the overexpression of *Irf4* also reduced BCL6 expression in CXCR5^+^PD-1^+^ cells relative to that in the empty transduction group (Fig. 5E).

On the other hand, we performed additional loss-of-function experiments via the *Cd4*-Cre^+^*Irf4*^fl/fl^ knockout mice previously used by our team [33]. Briefly, *Cd4*-Cre^+^*Irf4*^fl/fl^ knockout or *Cd4*-Cre^-^*Irf4*^fl/fl^ WT mice were immunized with SRBCs on day 0, followed by treatment with trametinib or an equivalent amount of corn oil by oral gavage daily from day 3 to day 6, and the spleens were harvested and analyzed by FCM on day 7(Fig. S6F). Notably, we observed that MEK inhibitor treatment significantly enhanced Tfh cell generation in wild-type mice, whereas it failed to induce Tfh cell differentiation in *Irf4*-knockout mice (Fig. S6G-H).

Collectively, these data indicated that both insufficient IRF4 activity and supra-physiological levels can disrupt the normal Tfh differentiation program, implying the non-linear phenotypic outcomes. Nevertheless, our data established a critical functional axis whereby MEK1/2 signaling promotes Tfh differentiation through IRF4. The genetic loss-of-function experiments demonstrated that IRF4 is an indispensable downstream effector of this pathway, as *Irf4* deficiency abrogated the Tfh phenotype. Notably, our data also suggested that the relationship between IRF4 activity and Tfh output may not be strictly linear, a concept consistent with observations by Krishnamoorthy et al. [14] showing that Tfh cells arise within an intermediate range of IRF4 expression. However, the central conclusion of our study remains that IRF4 serves as a necessary link between the MEK signaling cascade and the Tfh gene program.

### MEK1/2 inhibitors augment Tfh differentiation in vitro and in vivo

Because we revealed that T-cell-specific *Mek*1/2 deletion potentiates Tfh differentiation and that the GC B-cell response is dependent on IRF4 epigenetic modulation, we next sought to evaluate whether pharmacological inhibition of MEK1/2 can achieve a parallel facilitating effect on Tfh cell fate programming. To selectively inhibit MEK1/2 signaling, we used trametinib, which was approved by the Federal Drug Administration and the European Medicines Agency for the treatment of metastatic melanoma [69].

**Fig. 6:**
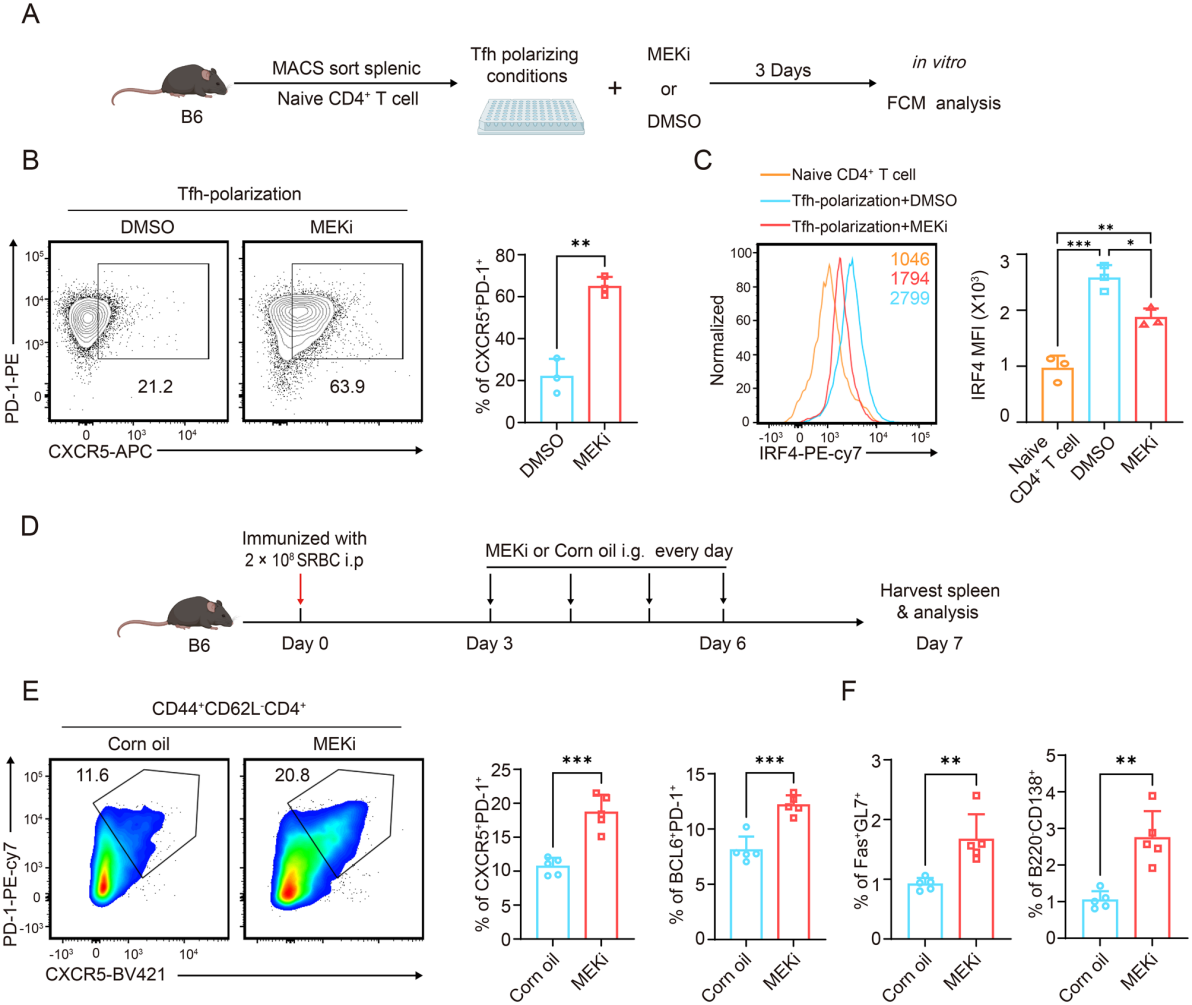
The MEK1/2 inhibitor trametinib augments Tfh differentiation in vitro and in vivo. (**A**) Schematic of in vitro Tfh polarization with *Mek*1/2 inhibitor treatment. (**B**) Representative contour plots and bar plots showing the percentages of Tfh (CXCR5^+^PD-1^+^ cells) cells (*n* = 3). (**C**) Representative histograms of IRF4 expression in the naïve CD4^+^ T-cell group, DMSO group and MEK1/2 inhibitor group. The mean fluorescence intensity (MFI) is summarized on the right (*n* = 3). (**D**) Schematic of in vivo SRBC immunization with *Mek*1/2 inhibitor treatment. (**E**) Representative psuedocolor plots and bar plots showing the percentages of CXCR5^+^PD-1^+^ and BCL6^+^PD-1^+^ cells among CD44^+^CD62L^-^CD4^+^ cells from SRBC-immunized B6 mice treated with trametinib or corn oil on day 7 (*n* = 5). (**F**) Representative psuedocolor plots and bar plots showing the percentages of Fas^+^GL7^+^ cells among B220^+^ cells and B220^-^CD138^+^ cells among CD3^-^CD11b^-^ cells from SRBC-immunized B6 mice treated with trametinib or corn oil on day 7 (*n* = 5). The data are representative of at least three independent experiments. The error bars represent the means ± SD; *p* values in (B,E,F) were calculated using an unpaired two-tailed t test; *p* values in (C) were calculated using one-way ANOVA followed by Tukey’s multiple-comparisons test; *, *p* < 0.05; **, *p* < 0.01; ***, *p* < 0.001; ****, *p* < 0.0001; ns, not significant

First, we performed in vitro Tfh polarization experiments with or without MEK1/2 inhibitor treatment. Naïve CD4^+^ T cells were sorted from the spleens of C57BL/6 mice and stimulated in 96-well plates (coated with anti-CD3 and anti-CD28 antibodies) plus anti-IL-2, anti–IFN-γ, anti-IL-4, anti-TGF-β and IL-6 to induce Tfh polarization. The medium was further supplemented with 0.2% DMSO or 100 nM trametinib (Fig. 6A). The cells were cultured for 3 days in vitro, followed by FCM analysis. As expected, MEK1/2 inhibitor treatment obviously diminished p-MEK1/2 and p-ERK1/2 expression in cultured CD4^+^ T cells under Tfh cell skewing conditions (Fig. S4, A-B). Like the percentage of *Mek*1/2-deficient CD4^+^ T cells, the percentage of Tfh (identified as CXCR5^+^PD-1^+^ cells) cells among the trametinib-treated polarized CD4+ T cells was significantly greater than that in the DMSO-treated group (Fig. 6B). Moreover, we also observed obviously elevated BCL6 expression in trametinib-treated polarized CD4+ T cells (Fig. S4C). Moreover, compared with DMSO, trametinib was sufficient to inhibit the expression of IRF4 in polarized CD4^+^ T cells (Fig. 6C). Overall, our findings demonstrated that the MEK1/2 inhibitor trametinib effectively decreased IRF4 expression in polarized CD4^+^ T cells and subsequently enhanced Tfh cell differentiation in vitro.

Next, we examined whether transient trametinib treatment effectively augmented Tfh development and the GC B-cell response in vivo. Briefly, WT C57BL/6 mice were immunized with SRBCs on day 0, followed by treatment with trametinib (0.3 mg/kg/day; resuspended in 10% DMSO with 90% corn oil) or an equivalent amount of corn oil by oral gavage daily from day 3 to day 6, and the spleens were harvested and analyzed by FCM on day 7 (Fig. 6D). After trametinib treatment, we detected significantly elevated percentages of CXCR5^+^PD-1^+^ and BCL6^+^PD-1+ cells among CD44^+^CD62L^-^CD4^+^ T cells upon SRBC immunization (Fig. 6E and Fig. S4D). Moreover, compared with those in the control group, the percentages of Fas^+^GL7^+^ cells among B220^+^ cells and B220^-^CD138^+^ cells among CD3^-^CD11b^-^ cells in the trametinib-treated group were also greater (Fig. 6F and Fig. S4, E-F). Together, these findings are in agreement with those of the Tfh polarization experiment in vitro, which revealed that the MEK1/2 inhibitor trametinib was sufficient to promote Tfh differentiation and the GC B-cell response after SRBC immunization in vivo.

In conclusion, these data indicate that the MEK1/2 inhibitor trametinib effectively facilitates Tfh cell development both in vitro and in vivo. Moreover, our results provide new insight into the potential of the MEK1/2 inhibitor trametinib as a possible agent for modulating T-cell development.

### MEK1/2 inhibitors enhance Tfh cell development coupled with humoral immune responses in a murine melanoma model

Through the above experiments, we demonstrated that genetic ablation or pharmacological inhibition of MEK1/2 has potential for the modulation of Tfh cell differentiation in therapeutic applications. Some previous studies have revealed favorable immunomodulatory effects of MEK1/2 inhibitor on the tumor microenvironment via various mechanisms [46]. However, most studies have focused on combination therapy strategies involving MEK1/2 inhibitor treatment and other antitumor therapies [70, 71]; very little is known about the impact of MEK inhibitor treatment on tumor-infiltrating immune cell function to realize the potential antitumor effects of MEK1/2 inhibitor. Thus, we next focused our study on how MEK inhibitor treatment influences immune cells in the TME. To characterize the heterogeneity of immune cells in the TME under MEK1/2 inhibitor treatment globally, we utilized a published murine scRNA-seq dataset (GSE177901) of melanoma tumor-infiltrating CD45^+^ immune cells derived from the untreated, MEK1/2 inhibitor-treated and MEK1/2 inhibitor plus anti-PD-L1 treatment groups [50]. After sample integration and quality control, 27,263 CD45^+^ cells sorted from the TME across 3 different treatments were further categorized into 7 major cell clusters. According to the expression profiles of cluster-defining signature genes (Fig. S4H), we separately annotated 7 major cell types, namely, CD4^+^ T cells, CD8^+^ T cells, B cells, myeloid cells, NK cells, fibroblasts, and DCs (Fig. S4G). Among these cell types, MEK1/2 inhibitor treatment and MEK1/2 inhibitor combined with anti-PD-L1 treatment predominantly increased the proportions of tumor-infiltrating CD4^+^ and CD8+ T cells (Fig. S4I), so we next focused on tumor-infiltrating T cells.

**Fig. 7:**
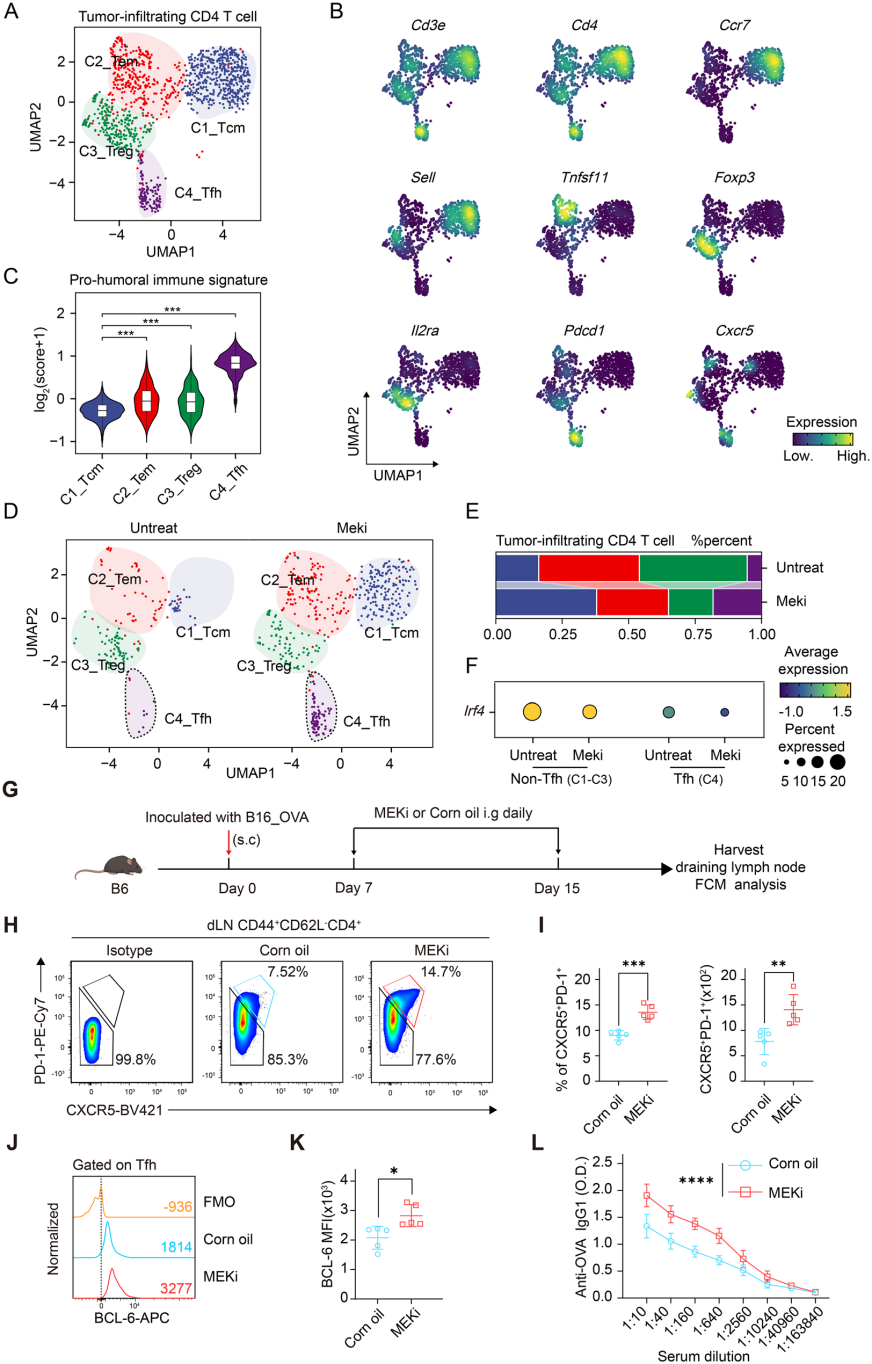
MEK1/2 inhibitor treatment drives Tfh cell development and humoral immune responses in a murine melanoma model. (**A**) UMAP plot showing the 4 subclusters generated from tumor-infiltrating CD4^+^ T cells from the published murine melanoma dataset GSE177901. (**B**) UMAP density plot showing the expression of cluster-defining signature genes. (**C**) Violin plot showing the pro-humoral immune response score across each CD4^+^ T-cell subcluster. (**D**) UMAP plot showing the dynamic changes in the 4 subclusters from tumor-infiltrating CD4^+^ T cells with or without MEK inhibitors treatment. (**E**) Stacked bar plot showing the percentages of each subcluster from CD4^+^ T cells with or without MEK1/2 inhibitor treatment, related to the UMAP plot in (D). (**F**) Dot plot showing the relative average expression of *Irf4* across CD4^+^ T-cell subclusters with or without MEK inhibitors treatment. (**G**) Schematic of the tumor experimental design. (**H**) Representative psuedocolor plots showing the percentages of CXCR5^+^PD-1^+^ (Tfh) cells among CD44^+^CD62L^-^CD4^+^ cells from recipient mice in the dLns on day 15 after trametinib or corn oil treatment. (**I**) Scatter plots showing the percentages and numbers of CXCR5^+^PD-1^+^ (Tfh) cells (*n* = 5). (**J**) Representative histograms of BCL6 expression among FMO, corn oil and trametinib group. FMO, fluorescence minus one. (**K**) Scatter plot showing the MFI of BCL6 (*n* = 5). (**L**) Detection of serum levels of anti-OVA-specific IgG1 by ELISA 15 days after B16_OVA inoculation. The data are representative of at least three independent experiments. The error bars represent the means ± SD; *p* values in (I, K) was calculated using unpaired two tailed t-test; *p* value in (L) was calculated using two-way ANOVA with šídák’s multiple comparison; *, *p* < 0.05; **, *p* < 0.01; ***, *p* < 0.001; ****, *p* < 0.0001

To assess the dynamics and diversity of the tumor-infiltrating CD4^+^ T-cell repertoire after MEK1/2 inhibitor treatment, we performed high-resolution reclustering analyses. Unsupervised clustering identified 4 subclusters (Fig. 7A) and characterized each subpopulation-specific transcriptional signature (Fig. 7B and Fig. S5A). Subcluster 1, termed central memory T cells (C1_Tcm), highly expressed *Tcf7*, *Ccr7*, and *Sell*. Subcluster 2, annotated as effector memory T cells (C2_Tem), presented similar memory signatures but presented higher levels of *Tnfsf11*, *Tbc1d4*, and *Itgb1*. Subcluster 3 (C3_Treg) cells were identified as regulatory T cells owing to the coexpression of *Foxp3* and *Il2ra*. Notably, although *Bcl6* expression was not detected well in the C4 subset, this may be due to the limited sensitivity of scRNA-seq. Subcluster 4 (C4_Tfh) could be separated from other subsets by the expression of *Pdcd1* and *Cxcr5*, which are characteristic markers for follicular helper T cells (Fig. S5A). Consistent with this clustering, the AddModuleScore function was applied to evaluate the pro-humoral immune response signature [72] across each CD4^+^ T subcluster, which further confirmed that the C4_Tfh subset tended to exhibit the features of follicular helper T cells (Fig. 7C). Moreover, through GO enrichment analysis of highly variable genes expressed in the C4_Tfh subset, we also found that many pathways associated with the immunological programs of follicular helper T cells, including those related to the regulation of B-cell activation, proliferation, and differentiation, were enriched (Fig. S5D). Moreover, more C4_Tfh cells infiltrated the TME after MEK inhibitor treatment (Fig. 7D–E), whereas supplementation with anti-PD-L1 treatment did not further improve the infiltration of follicular helper T cells (Fig. S5B). As expected, the increased proportion of TME-infiltrating C4_Tfh cells was accompanied by decreased *Irf4* transcription in the C4_Tfh subset following MEK1/2 inhibitor treatment, suggesting that MEK1/2 inhibitor-mediated Tfh cell infiltration may rely on the potential regulator IRF4 (Fig. 7F). Taken together, these bioinformatics analysis results suggest that MEK1/2 inhibitor treatment enhances Tfh cell infiltration in the TME of mouse melanoma in an IRF4-dependent manner.

To validate the above scRNA-seq observations, we employed a mouse B16_OVA melanoma model and administered inhibitor intervention (Fig. 7G). Briefly, WT C57BL/6 mice were inoculated with B16_OVA tumors on day 0, followed by treatment with trametinib (0.3 mg/kg/day; resuspended in 10% DMSO with 90% corn oil) or an equivalent amount of corn oil by oral gavage daily from day 7 to day 15 (Fig. 7H). On day 15 after MEK1/2 inhibitor therapy, we evaluated Tfh cell infiltration in the draining lymph nodes of tumor-bearing mice. In line with the findings obtained from the scRNA-seq data, we detected markedly increased percentages and numbers of CXCR5^+^PD-1^+^ Tfh cells among the CD44^+^CD62L^-^CD4^+^ T cells after trametinib therapy (Fig. 7I). Meanwhile, the MFI of BCL6 among Tfh cells in the trametinib-treated group was also elevated relative to that in the corn oil control group (Fig. 7, J and K). Consistent with the enhanced Tfh cell development, OVA-specific IgG1 levels in the serum of trametinib-treated tumor-bearing mice were also obviously elevated (Fig. 7L). Overall, we found that MEK1/2 inhibitor treatment increased the proportion of draining lymph node and tumor-infiltrating Tfh cells, coupled with a stronger humoral immune response in a murine melanoma model.

To further clarify the link between Tfh expansion, humoral immunity, and antitumor efficacy, we performed complementary experiments involving both B cell transfer and depletion. For the B-cell transfer assay, *Rag1*^-/-^ mice were firstly inoculated with B16_OVA tumor at day0 and were immunological reconstituted with OT-II T cells plus B cells at day5, followed by treated with trametinib or equivalent Corn oil by oral gavage daily from day5 to day20, tumor dLNs were harvested and measured by FCM (Fig. S7A). On day12, we detected markedly increased percentages of CXCR5^+^PD-1^+^ and BCL6^+^PD-1^+^ cells among CD44^+^CD62L^-^CD4^+^ T cells post trametinib therapy (Fig. S7C). Similarly, the percentages of Fas^+^GL7^+^ cells among B220^+^cells and B220^-^CD138^+^cells among CD3^-^CD11b^-^cells in trametinib-treated group were also elevated relative to control group (Fig. S7D). Moreover, results showed that MEK1/2 inhibitor treatment in OT-II; cells and B cells significantly improved B16_OVA tumor controls in transferred recipients (Fig. S7B). For the B-cell depletion assay, following administration of 250 μg anti-mouse CD20 antibody one day prior, C57BL/6 mice were inoculated with B16F10 tumors on day 0 and then underwent daily oral gavage with either trametinib or a corn oil vehicle control from day 5 to day 20 (Fig. S7E). We observed that MEK inhibitor treatment significantly suppressed tumor growth. Notably, this inhibitory effect partially disappeared when B cells were depleted (Fig. S7F). Taken together, these findings suggested that the antitumor efficacy of MEK inhibitors is partially mediated by their ability to promote humoral immune responses in a murine melanoma model, although additional mechanisms are likely involved.

### MEK inhibitors potentiate the humoral immune response and are correlated with favorable clinical outcomes in melanoma patients

**Fig. 8:**
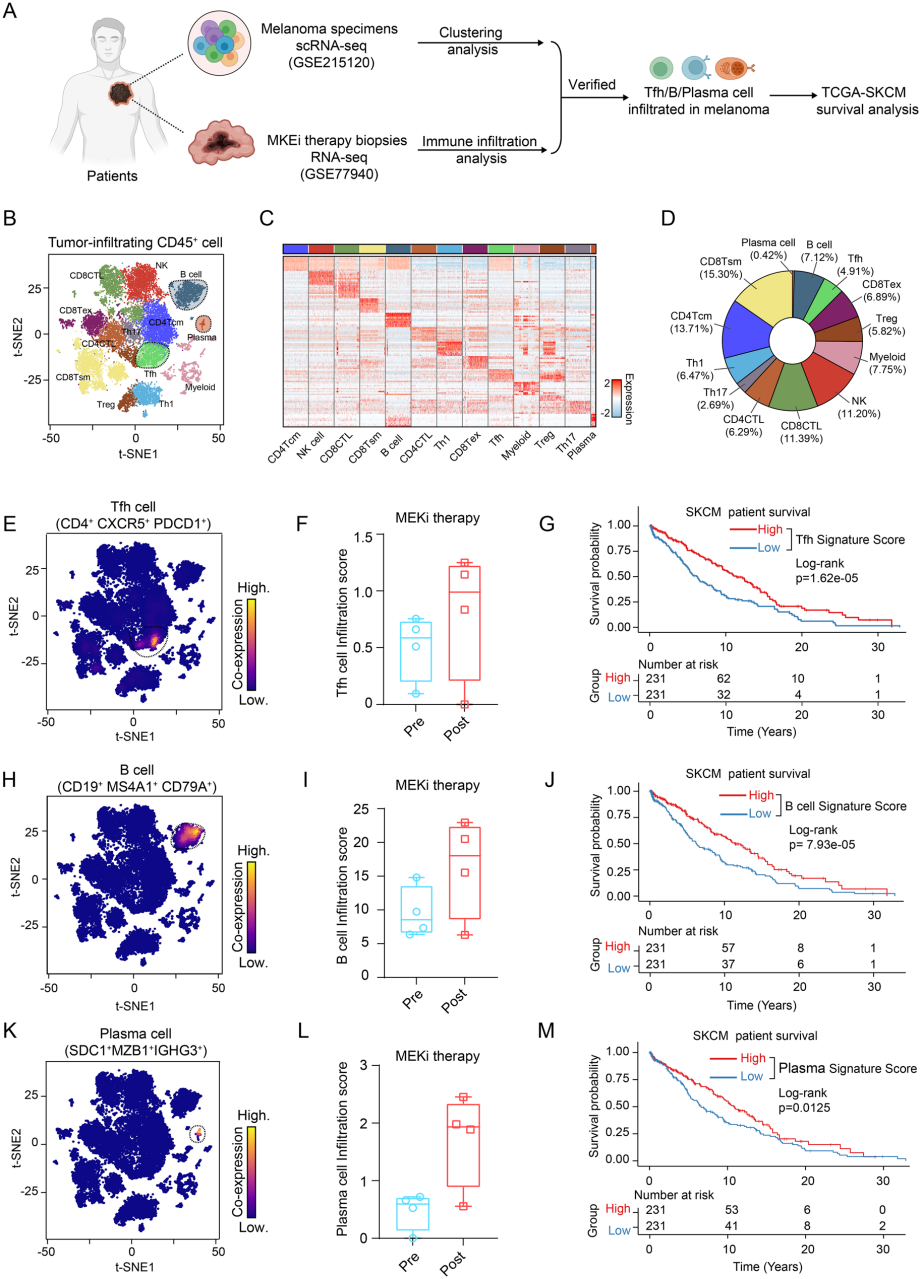
MEK1/2 inhibitor therapy potentiates the humoral immune response and is correlated with a favorable clinical prognosis in melanoma patients. (**A**) Overview of the analysis workflow. (**B**) TSNE plot showing major cell types generated from tumor-infiltrating CD45^+^ immune cells from the biopsy samples of melanoma patients (GSE215120). (**C**) Heatmap showing the normalized expression of differentially expressed genes (rows) among each cluster (columns). (**D**) Pie chart showing the percentages of each cluster of CD45^+^ immune cells. (**E**) TSNE density plot showing the coexpression level of Tfh cell cluster-defining signature genes. (**F**) Box plot showing the Tfh cell infiltration scores in pre- versus post-MEK1/2 inhibitor-treated tumor biopsies obtained from 4 melanoma patients (GSE77940), as calculated via the CIBERSORT algorithm. (**G**) Kaplan‒Meier plot showing the survival probability of patients with melanoma (from TCGA-SKCM data), stratified by expression levels of the Tfh cell signature score. (**H**) TSNE density plot showing the coexpression levels of B-cell cluster-defining signature genes. (**I**) Box plot showing the B-cell infiltration scores of pre- versus post-MKEi-treated tumor biopsies obtained from 4 melanoma patients (GSE77940), as calculated via the CIBERSORT algorithm. (**J**) Kaplan‒Meier plot showing the survival probability of patients with melanoma (from TCGA-SKCM data) stratified by the expression level of the B-cell signature score. (**K**) TSNE density plot showing the coexpression levels of plasma cell cluster-defining signature genes. (**L**) Box plot showing the plasma cell infiltration scores of pre- versus post-MEK1/2 inhibitor-treated tumor biopsies obtained from 4 melanoma patients (GSE77940), as calculated via the CIBERSORT algorithm. (**M**) Kaplan‒Meier plot showing the survival probability of patients with melanoma (from TCGA-SKCM data) stratified by the expression levels of the plasma cell signature score

Given that MEK1/2 inhibitors are widely used in clinical treatment [73], we next assessed the correlation between the main cellular subpopulations involved in the humoral immune response and the clinical features of melanoma patients (Fig. 8A). First, by analyzing the scRNA-seq dataset of tumor-infiltrating CD45^+^ immune cells from melanoma patient biopsies (GSE215120) (Fig. S5E) [74], we observed the prevalent infiltration of the major cellular components of the humoral immune response, including 4.91% T follicular helper (Tfh) cells, 7.12% B cells, and 0.42% plasma cells (Fig. 8, B -D). Consistent with the clustering results, our analysis of the coexpression levels of key signature genes across the three clusters further confirmed their distinct functions (Fig. 8, E and H and K). We subsequently performed immune infiltration analysis of tumor biopsy Bulk RNA-seq data (GSE77940) (Fig. S5F) [75], which showed that the infiltration scores of all three subpopulations were significantly increased following MEK1/2 inhibitor treatment (Fig. 8, F and I and L). Finally, we selected the marker genes of the three identified subpopulations from the aforementioned scRNA-seq datasets to calculate cell type-specific infiltration scores in the TCGA-SKCM cohort. Notably, survival analysis via Kaplan‒Meier curves with the log-rank test demonstrated a statistically significant association between increased infiltration of the three subpopulations and improved overall survival in melanoma patients (Fig. 8, G and J and M). Collectively, these clinical omics data suggest a potential association between MEK1/2 inhibitors and humoral immune responses, with the intensity of humoral immunity correlating with favorable clinical outcomes in melanoma patients.

## Discussion

In this work, we found that T-cell-specific *Mek*1/2 ablation potentiated Tfh differentiation in vitro and in vivo. Furthermore, omics analysis indicated that IRF4-dependent epigenetic modulation of Tfh cell-characteristic genes might serve as the driving force triggering the Tfh transcriptional program, which is responsible for T-cell-specific *Mek*1/2 deficiency-mediated Tfh differentiation. More importantly, our findings suggest that MEK1/2 inhibitor therapy enhances the Tfh-cell-dependent humoral response in a murine melanoma model and is correlated with a favorable clinical prognosis in melanoma patients.

It has been widely reported that MEK1/2 signaling is rapidly activated downstream of the TCR to drive multistage phosphorylation, thereby modulating several T-cell processes [26]. Shindo et al. reported that the RAS-MEK-ERK pathway is preferentially activated in naïve and central memory human T cells and that MEK inhibitors preferentially inhibit alloreactive T cells in graft-versus-host disease [28]. In addition, Wei et al. reported that upon T-cell activation, the MEK-ERK axis is robustly activated, thereby promoting glycolysis to ensure the appropriate proliferation of tilapia T cells [76]. Similarly, another research group reported that TCR stimulation reduces miR-15a/16 levels during the early stages of T-cell activation to facilitate increased MEK1 and ERK1, which promotes the sustained MEK1–ERK1/2-Elk1 signaling required for optimal proliferation [77]. Our previous study revealed that tramatinib, a selective inhibitor of Mek1/2, was sufficient to inhibit Th1 and Th17 cell differentiation and prolong heart allograft survival in acute cardiac allograft rejection [33]. In this study, similar results were obtained: T-cell-specific *Mek*1/2 ablation downregulated the expression of T-cell-characteristic transcription factors (*Gata3*, *Bhlhe40,* and *Nr4a3*) and cell surface molecules (*Il2ra*, *Il7r,* and *Ccr7*). Our in vitro polarization data show that MEK1/2 deficiency impairs Th1/Th17 differentiation while promoting Treg generation. Given the competitive relationships among CD4^+^ T cell subsets, enhanced Tfh differentiation may partly reflect passive redistribution from suppressed alternative fates, rather than active Tfh instruction. Conversely, MEK1/2 signaling might directly constrain Tfh programming. Current data cannot distinguish these non-mutually exclusive mechanisms. Future studies using lineage tracing, single-cell trajectory, and selective blockade of competing fates are needed. Nonetheless, our findings establish MEK1/2 as a critical regulator of CD4^+^ subset balance with functional consequences for Tfh differentiation and antitumor immunity. More remarkably, by using *CD4-Cre*^+^*Map2k1*^fl/fl^*Map2k2*^fl/fl^ double-gene knockout mice and *Mek*1/2-tKO OT-II transgenic mice, we confirmed that T-cell-specific *Mek*1/2 ablation potentiated Tfh differentiation and GC-B responses in a cell-intrinsic manner. In line with our findings, Wan et al. reported that the ERK kinase negatively regulates the Tfh cell differentiation program and that ERK2 inhibition enhances Tfh cell development in vitro and in vivo. However, Houde et al. reported that the kinases MEK1 and MEK2 play distinct roles in lymphocyte activation and that fine-tuning of MEK signaling is pivotal for limiting B- and T-cell activation [62]. Taken together, these results indicate that the precise mechanism by which MEK1/2 signaling modulates the T-cell phenotype is complicated and still requires further investigation.

IRF4 has emerged as a central determinant of *T*- and B-cell activation and differentiation, and its expression level varies with the intensity of antigen receptor signaling [32]. Here, through RNA-seq combined with ATAC-seq analysis, we revealed that *Mek*1/2 ablation reduced the expression of the transcription factor IRF4, which further enabled the epigenetic modulation of Tfh and non-Tfh cell-characteristic genes.While our ChIP data revealed reduced H3K4me3 at the *Irf4* promoter upon Mek1/2 deletion, this finding should be interpreted as evidence of an indirect regulatory link between MEK1/2 signaling and the epigenetic landscape. Determining whether this involves direct phosphorylation of histone-modifying enzymes by ERK or other downstream effectors will require further biochemical investigation. In a previously published study, Schmitt et al. suggested that IRF4 seems important in tipping the balance of Tfh and Th1 differentiation toward Tfh cells during the early stages [65]. In addition, Bollig et al. reported that IRF4 is an essential T-cell factor for Tfh cell differentiation and that Irf4^-/-^ mice fail to generate GCs [78]. Moreover, by employing an integrative genome viewer, we observed higher chromatin accessibility peaks at the partial and intact AICE loci among the key Tfh cell genes *Bcl6, Cxcr5* and *Bcl11b,* whereas increased chromatin accessibility peaks were observed at the potential ISRE loci in the non-Tfh key gene *Prdm1*. These results suggest that *Mek*1/2 ablation reduces the expression of IRF4, which preferentially binds to potential high-affinity recognition elements AICE at Tfh cell-characteristic gene loci and enhances Tfh development. In line with a previous study, Krishnamoorthy et al. demonstrated that Tfh cells arise in a bimodal manner if intermediate amounts of IRF4 are expressed, whereas Teff cell fate trajectories are linearly related to increasing amounts of IRF4 [14]. Optimal Tfh differentiation appears to require a specific range or threshold of IRF4 activity. Similarly, Saito et al. reported that higher levels of IRF4 bind to the BCL6 promoter region, thereby repressing BCL6 expression in B-cell lymphoma [79]. Overall, in this work, we described a conserved MEK1/2-IRF4 axis in Tfh cells that controls their cell fate dynamics, although we cannot completely exclude the possibility that other unidentified mechanisms are involved.

Over the past few decades, impressive advances in cancer therapy have significantly improved survival outcomes in cancer patients. Among these, small-molecule MKE inhibitors suppress tumor growth by selectively targeting oncogenic RAS_MAPK pathways [73]. However, despite these promising advancements, enormous challenges remain in achieving widespread efficacy across different cancer types. In particular, very little is known about how MEK inhibitors therapy influences tumor-infiltrating immune cells. In this study, through scRNA-seq analysis and tumor experiments, we observed that MEK inhibitors treatment suppressed IRF4 expression in Tfh cells, concomitant with enhanced Tfh cell recruitment to draining lymph nodes and tumor beds, ultimately driving humoral immune responses in a murine melanoma model. The partial reversal of tumor control upon B-cell depletion indicates that while humoral immunity contributes to the therapeutic efficacy of MEK inhibition, it likely operates in concert with other mechanisms—such as direct effects on tumor cells, modulation of the tumor microenvironment, or enhancement of T cell responses—to achieve maximal antitumor effect. Future studies combining MEK inhibition with B-cell-targeted or other immunomodulatory therapies may help delineate the relative contributions of these pathways. Furthermore, by integrating patient-derived biopsy profiling with TCGA cohort analysis, we demonstrated that MEK1/2 inhibitor-mediated infiltration of Tfh cells into melanoma was significantly correlated with improved clinical outcomes in patients. Similarly, Verma and colleagues reported that MEK1/2 inhibition through an epimetabolic-dependent mechanism reprogrammed CD8^+^ T cells into a memory stem phenotype with potent antitumor effects [30]. These findings suggest that the metabolic mechanisms that may be involved in the MEKi-driven Tfh-dependent humoral immune response are worthy of attention. Consistent with our findings, Fitzsimons et al. observed intratumoral B and plasma cells at the comprehensive pancancer single-cell level [36]. Moreover, Chaurio et al. revealed that Satb1 governs Tfh cell differentiation, leading to the assembly of tertiary lymphoid structures within tumors [42]. Thus, MEKi-driven Tfh and B-cell recruitment may orchestrate tertiary lymphoid structure assembly in tumor beds, an interesting question that warrants further investigation.

There are several limitations of this research. (1) Given that genetic ablation of *Mek*1/2 partially impairs T-cell development in the thymus, we generated *Mek*1/2-tKO OT-II^+^ mice and employed an adoptive transfer strategy to explore the T-cell-specific role of *Mek*1/2 in regulating Tfh-cell development. Therefore, subsequent studies are needed to more specifically evaluate the function of *Mek*1/2 signaling in Tfh cells by using an inducible *Mek*1/2 genetic ablation mouse strain. (2) Limited by experimental equipment, our current study principally depended on genetic ablation and in vitro assays. Considering the plasticity and diversity of Tfh cell development in time and space, in vivo and in situ real-time detection of MEK1/2 phosphorylation may provide more compelling evidence of our results. (3) An important consideration when interpreting the enhanced Tfh phenotype upon *Mek1/2* deletion is whether this reflects active instruction toward the Tfh lineage or, alternatively, a passive consequence of lineage redistribution. Our in vitro polarization data reveal that MEK1/2 deficiency simultaneously impairs Th1 and Th17 differentiation while promoting Treg generation. Thus, it is plausible that the increased Tfh frequency observe d under Tfh-polarizing conditions may partly result from a shift in the equilibrium of lineage decisions. In this model, MEK1/2 signaling normally restrains Tfh differentiation in part by supporting alternative fates (Th1/Th17); its absence reduces the ‘pull’ toward these lineages, allowing more cells to adopt the Tfh or Treg programs favored by the cytokine milieu. While our data demonstrated that MEK1/2 signaling is a critical regulator of CD4^+^ subset differentiation, the precise mechanism—whether direct lineage instruction or indirect redistribution—likely involves both components and will require more granular fate-mapping or competitive systems to fully disentangle. (4) Mechanistically, based on the integrative analysis of RNA-seq and ATAC-seq data, we focused on whether IRF4 acts as a key regulator in shaping Tfh cell differentiation, but whether other important molecules downstream of *Mek*1/2 are involved is still not completely clear. While our data established an epigenetic association between MEK1/2 signaling and IRF4 expression, the precise biochemical mechanisms linking MEK/ERK to chromatin regulation remain to be elucidated. Future studies employing phosphoproteomic screening, in vitro kinase assays, and genetic models targeting candidate chromatin modifiers will be necessary to identify direct ERK substrates in Tfh cells and determine whether ERK-mediated phosphorylation events directly govern *Irf4* transcription. (5) While our B cell transfer and depletion experiments established that humoral immunity is functionally required for the anti-tumor efficacy of MEK inhibition, they do not formally prove that Tfh cells are the indispensable upstream mediators. MEK inhibitors could potentially act directly on B cells or through other helper subsets to influence humoral responses. The definitive demonstration of Tfh necessity would require Tfh-specific perturbations such as CD4-Cre Bcl6 deletion, CXCR5 blockade, or ICOSL disruption in the context of MEK inhibition. However, the concordant observation that MEK inhibition expands Tfh cells, enhances germinal center B cell and plasma cell responses, and confers B cell-dependent tumor control—together with the established paradigm that Tfh cells are the primary drivers of T-dependent humoral immunity—strongly suggests that the anti-tumor effects are mediated, at least in part, through the Tfh-B cell axis. Future studies employing Tfh-specific genetic models will be necessary to definitively establish this causal relationship. (6) Due to the limited number of clinical samples from MEK1/2 inhibitor therapy cohorts, subsequent studies will collect more patient samples to comprehensively evaluate the modulatory effects of MEK inhibitors on the antitumor humoral response, with a specific focus on its role in tertiary lymphoid structure. Nonetheless, our results suggest that the MEK1/2-IRF4 axis potentiates Tfh development to enhance antitumor humoral responses.

## Conclusions

**Fig. 9:**
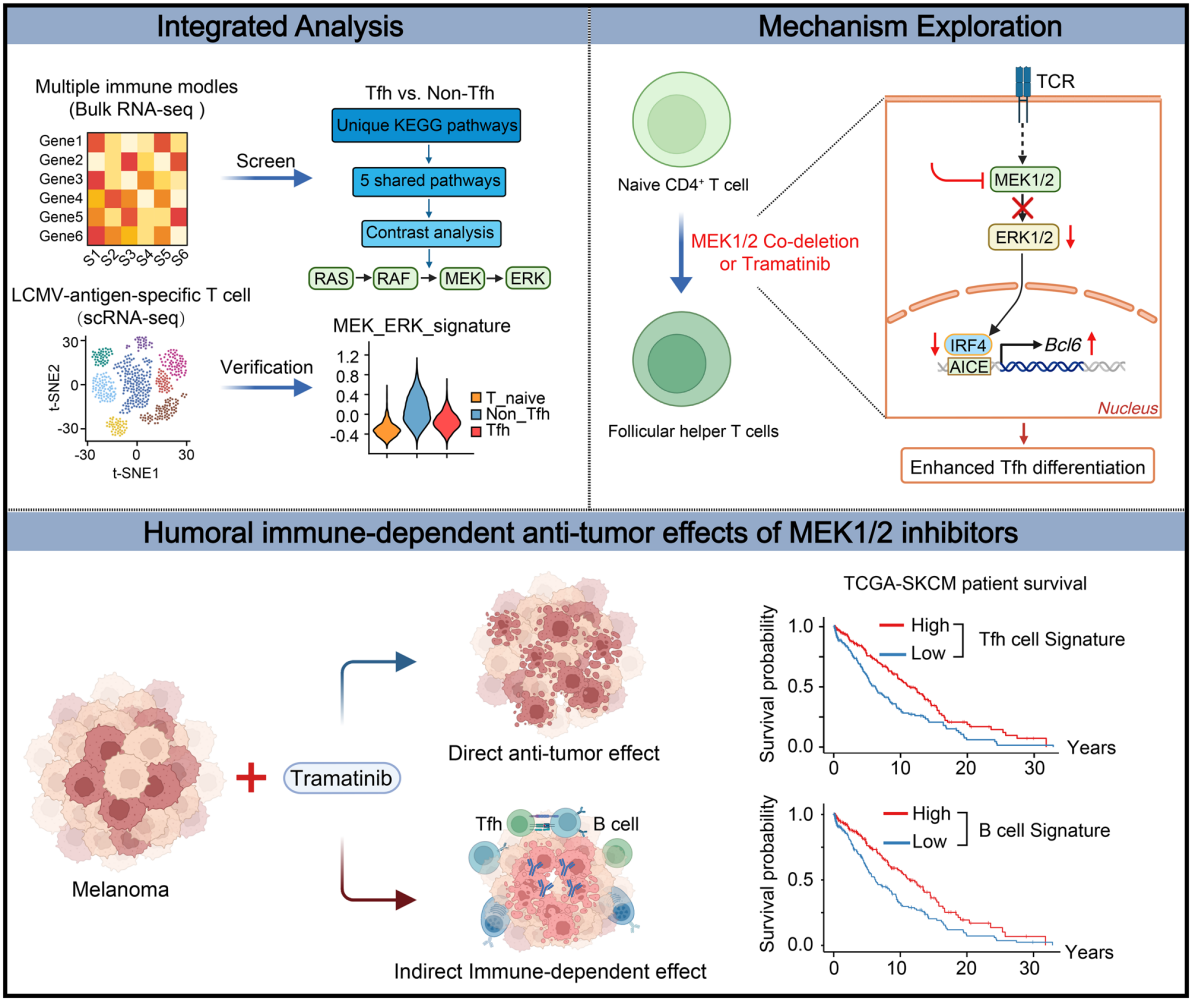
Graphical abstract. Integrated analysis of Tfh cells from multiple immune models revealed that the MEK1/2 signaling pathway is differentially activated in Tfh and non-Tfh cells in the context of various immune responses. Experiments involving gene ablation or pharmacological inhibition of MEK1/2 confirmed that the MEK1/2-IRF4 axis plays a crucial role in the regulation of Tfh development. Targeting MEK1/2 enhances the efficacy of antitumor immunotherapy by modulating Tfh-cell-dependent humoral responses and is associated with a favorable clinical prognosis in melanoma patients. The graphical abstract was generated by BioRender.com. Tfh, T follicular helper; MEK1/2, mitogen-activated protein kinase kinase 1/2; IRF4, interferon regulatory factor 4; *Bcl6*, B-cell leukemia/lymphoma 6; AICE, AP-1-IRF composite elements

In summary, our current study described the MEK1/2-IRF4 axis, which plays a crucial role in governing Tfh development in a T-cell-intrinsic manner. Moreover, our data linked MEK1/2 inhibitor-driven Tfh cell infiltration to melanoma immune microenvironment remodeling, where heightened humoral responses were positively correlated with a favorable clinical prognosis (Fig. 9). These findings may provide an unprecedented strategy to improve antitumor immunotherapy efficacy by harnessing humoral immune responses.

## Electronic supplementary material

Below is the link to the electronic supplementary material.

Supplementary material 1

## Data Availability

The RNA-seq and ATAC-seq data reported in this article have been deposited in the Gene Expression Omnibus (GEO) database under accession number GSE299327, GSE299522 and GSE299628. Any additional information and materials are available from the lead contact upon reasonable request.

## Abbreviations

TME: Tumor immune microenvironment
TIL: Tumor-infiltrating lymphocytes
dLN: Draining lymph nodes
TLS: Intratumoral tertiary lymphoid structures
GC: Germinal center
Tfh: T follicular helper cell
MEK1/2: Mitogen-activated protein kinase kinase 1/2
IRF4: Interferon regulatory factor 4
BCL6: B-cell leukemia/lymphoma 6
AICE: AP-1-IRF composite elements
LCMV: Lymphocytic choriomeningitis virus
SRBC: Sheep red blood cells
OVA: Chicken ovalbumin
CFA: Complete Freund’s adjuvant
FCM: Flow cytometry analysis
MFI: Mean fluorescence intensity
MEKi: MEK1/2 inhibitor
TCGA: The Cancer Genome Atlas
SKCM: Skin cutaneous melanoma
GEO: Gene Expression Omnibus
GSEA: Gene set enrichment analysis
GO: Gene Ontology
ChAR: Chromatin-accessible regions
RNA-seq: RNA sequencing
scRNA-seq: Single-cell RNA sequencing
ATAC-seq: Assay for Transposase-Accessible Chromatin with high throughput sequencing

## Code availability

All data analyses in this study were conducted via publicly available codes and open source packages. No original codes or new algorithms were generated in this study.
