# Supplementary material for: The MEK1/2-IRF4 axis fosters T follicular helper cell differentiationand antitumor humoral immune response
Source: J Transl Med. 2026 Mar 16;24:576. doi: 10.1186/s12967-026-08023-2 (PMC13104427; doi:10.1186/s12967-026-08023-2)
Supplement: Supplementary file 1 — Supplementary material 1 [file 12967_2026_8023_MOESM1_ESM.docx]

Supplemental material for

**The MEK1/2-IRF4 axis fosters T follicular helper cell differentiation and antitumor humoral immune response**

Shuan Ran^1,2^†, Song Wang^1,2^†, Ran Li^1,2^, Longyong Lai^1,2^, Jizhang Yu^1,2^, Xi Zhang^1,2^, Yuan Li^1,2^, Weicong Ye^1,2^, Junjie Zong^1,2^, Xiaohan Li^1,2^, Yanglin Hao^1,2^, Jiulu Zhao^1,2^, Zilong Luo^1,2^, Han Zhang^1,2^, Kexiao Zheng^1,2^, Pinyan Huang^1,2^, Wang Zhan^1,2^, Zifeng Zou^1,2^, Yanqiang Zou^1,2^, Jikai Cui^1,2,3*^, Jie Wu^1,2,3*^, Jiahong Xia^1,2,3*^

**Affiliations:**

^1^Department of Cardiovascular Surgery, Union Hospital, Tongji Medical College, Huazhong University of Science and Technology, Wuhan, China

^2^Center for Translational Medicine, Union Hospital, Tongji Medical College, Huazhong University of Science and Technology, Wuhan, China

^3^Key Laboratory of Organ Transplantation, Ministry of Education; NHC Key Laboratory of Organ Transplantation; Key Laboratory of Organ Transplantation, Chinese Academy of Medical Sciences, Wuhan, China

†These authors contributed equally to this work.

^*^Corresponding author. Email:

jiahong.xia@hust.edu.cn (J.X.); wujie426@hust.edu.cn (J.W.); jacobcjk@163.com (J.C.)

**This supplementary material PDF file includes:**

Figure S1 to S7


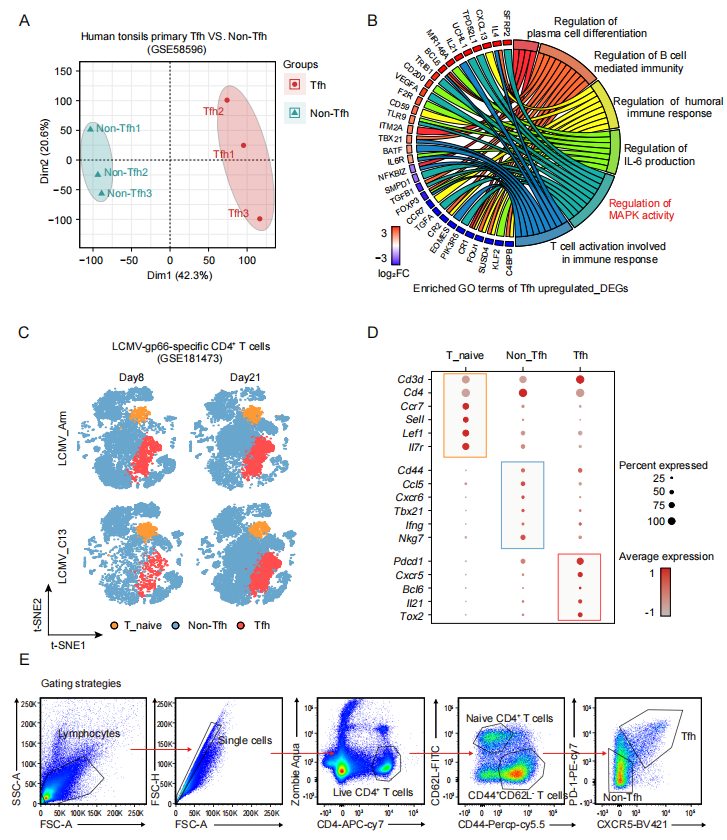


**Figure S1.** Bioinformatics analysis of the GEO public datasets and FCM analysis of the SRBC immunization model, related to Figure 1.

(A) Principal component analysis (PCA) of RNA-seq profiles from human tonsillar primary Tfh and non-Tfh cells (derived from published GEO dataset GSE58596). Each dot represents one biological replicate. (B) Chord plot representing the enriched Gene Ontology (GO) pathways belonging to the Biological Process subontology based on Tfh cells upregulated DEGs. Chords show the relationship between the log_2_-fold change (log_2_FC) of DEGs (left semicircle) and their enriched GO terms (right semicircle). (C) TSNE plot showing the dynamic change of 3 sub-clusters from murine LCMV-gp66-specific CD4^+^ T cells at indicated time points under two different conditions. (D) Dot plot showing the relative average expression of cluster-defining signature genes (y axis) across the clusters (x axis). (E) Gating strategy for identification of Tfh and Non-Tfh in splenocytes of SRBC immunized mice at day 7.


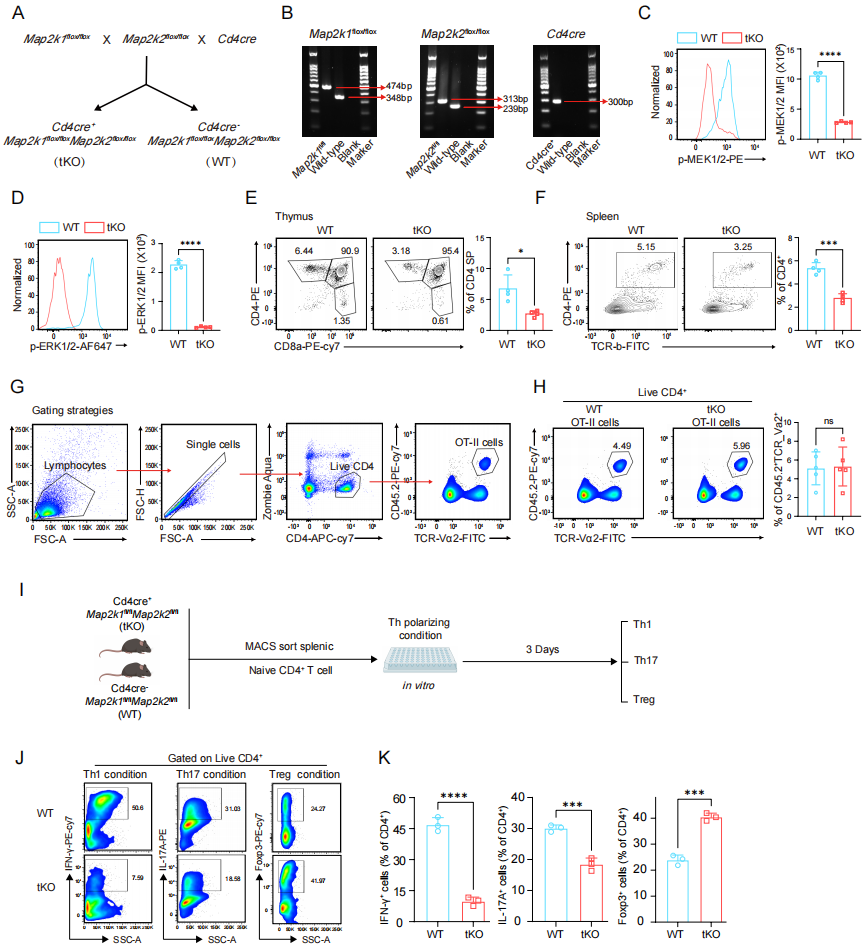


**Figure S2.**  Genotypic and phenotypic analysis of the T-cell-specific *Mek*1/2 knockout mice, related to Figure 2 and Figure 3.

(A) Schematic of the construction strategy for T-cell-specific *Mek*1/2 knockout mice. (B) Genotypes of *Mek*1/2-knockout (*Mek*1/2-tKO) mice were identified by PCR of tail DNA, representative southern blotting images showing the expression of *Map2k1*^flox/flox^, *Map2k1*^flox/flox^ and *Cd4-cre*. (C) Representative histograms of p-MEK1/2 in WT and *Mek*1/2-tKO were shown, MFI were depicted alongside (n=4). (D) Representative histograms of p-ERK1/2 in WT and *Mek*1/2-tKO were shown, MFI were depicted alongside (n=4). (E) Representative contour plots and bar plots of the percentages of CD4 single positive (CD4^+^CD8a^-^) cells among thymic cells from WT or *Mek*1/2-tKO mice (n=4). (F) Representative contour plots and bar plots of the percentages of CD4^+^TCRb^+^ cells among Splenic cells from WT or *Mek*1/2-tKO mice (n=4). (G) Gating strategy for identification of OT-II (CD45.2^+^TCR-Va2^+^) T cells in dLNs of OVA/CFA immunized mice at day 7. (H) Representative psuedocolor plots showing the percentages of OT-II (CD45.2^+^TCR-Va2^+^) T cells among live CD4^+^ T cells in dLNs of OVA/CFA immunized WT or *Mek*1/2-tKO OT-II^+^ mice at day 7. Statistic was depicted alongside (n=5). (I) Schematic of *in vitro* Th polarization. (J) Representative psuedocolor plots showing the percentages of IFN-γ^+^ cells, IL-17A^+^ cells and Foxp3^+^ cells among live CD4^+^ cells from WT or *Mek*1/2-tKO mice on day 3 post-polarization (n=5). (K) Representative bar plots of the percentages of IFN-γ^+^ cells, IL-17A^+^ cells and Foxp3^+^ cells among live CD4^+^ cells from WT or *Mek*1/2-tKO mice on day 3 post-polarization (n=5). Data were representative of at least three independent experiments. Error bars show Mean ± SD; P values in (C-E) were calculated using unpaired t-test with welch’s correction; P values in (F,H,K) were calculated using Unpaired two tailed t-test; *, P < 0.05; **, P < 0.01; ***, P < 0.001; ****, P < 0.0001; ns, no significance.


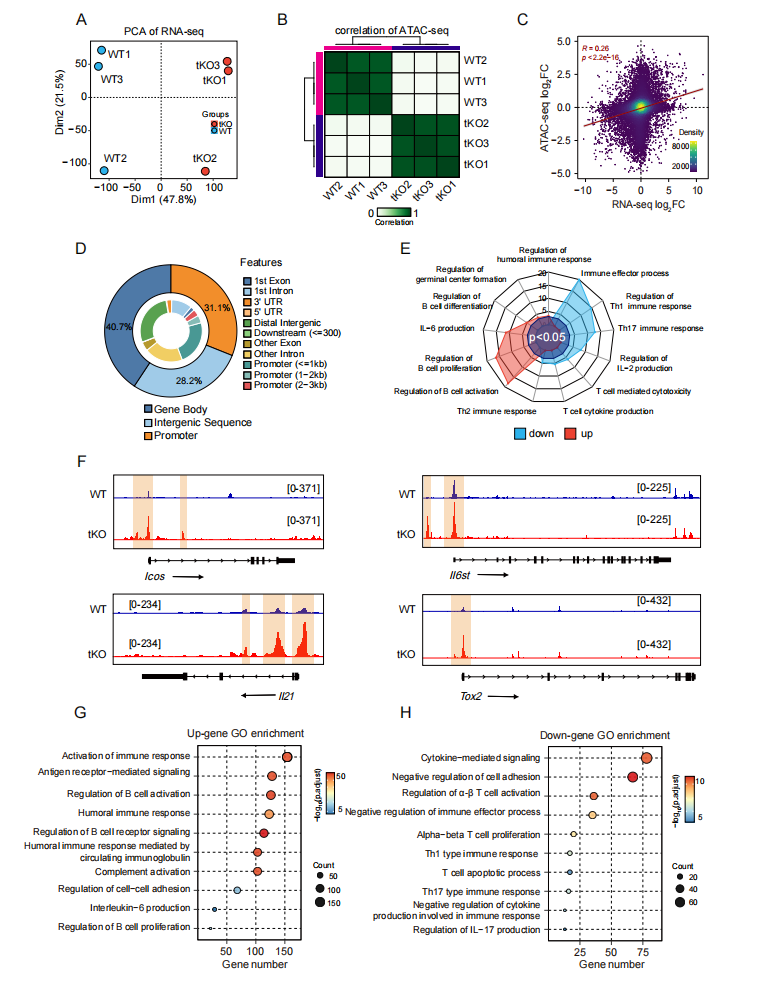


**Figure S3.** Additional details of combined analysis of RNA-seq and ATAC-seq , related to Figure 4.

Principal component analysis (PCA) of the RNA-seq profiles based on adoptively transferred CD45.2^+^ *Mek*1/2-tKO versus WT OT-II T cells at day7 post-OVA/CFA immunization, each dot represents one biological replicate. (B) Pearson’s correlation analyses of ATAC-seq samples between adoptively transferred CD45.2^+^ *Mek*1/2-tKO and WT OT-II T cells at day7 post-OVA/CFA immunization. (C) Scatter plot depicting the coincident transcriptional and epigenomic changes associated with T-cell-specific *Mek*1/2 deletion in CD45.2^+^ OT-II T cells at day7 post-OVA/CFA immunization. (D) Pie chart depicting the genomic distribution of differentially-accessible chromatin regions in adoptively transferred CD45.2^+^ *Mek*1/2-tKO versus WT OT-II T cells upon OVA/CFA immunization. (E) Radar plot showing the degree of enrichment of representative pathways for the Up and Down-gene identified from figure 4F using enrichment analysis. (F) Integrative genome viewer depicting the representative ATAC-seq signal tracks in the Tfh and Non-Tfh cell-characteristic genes locus from *Mek*1/2-tKO versus WT CD45.2^+^OT-II T cells. (G) Dot plot showing the significant GO pathways among the upregulated genes of transferred *Mek*1/2-tKO CD45.2^+^OT-II T cells at day7 post-OVA/CFA immunization. (H) Dot plot showing the significant GO pathways among the downregulated genes of transferred *Mek*1/2-tKO CD45.2^+^OT-II T cells at day7 post-OVA/CFA immunization.


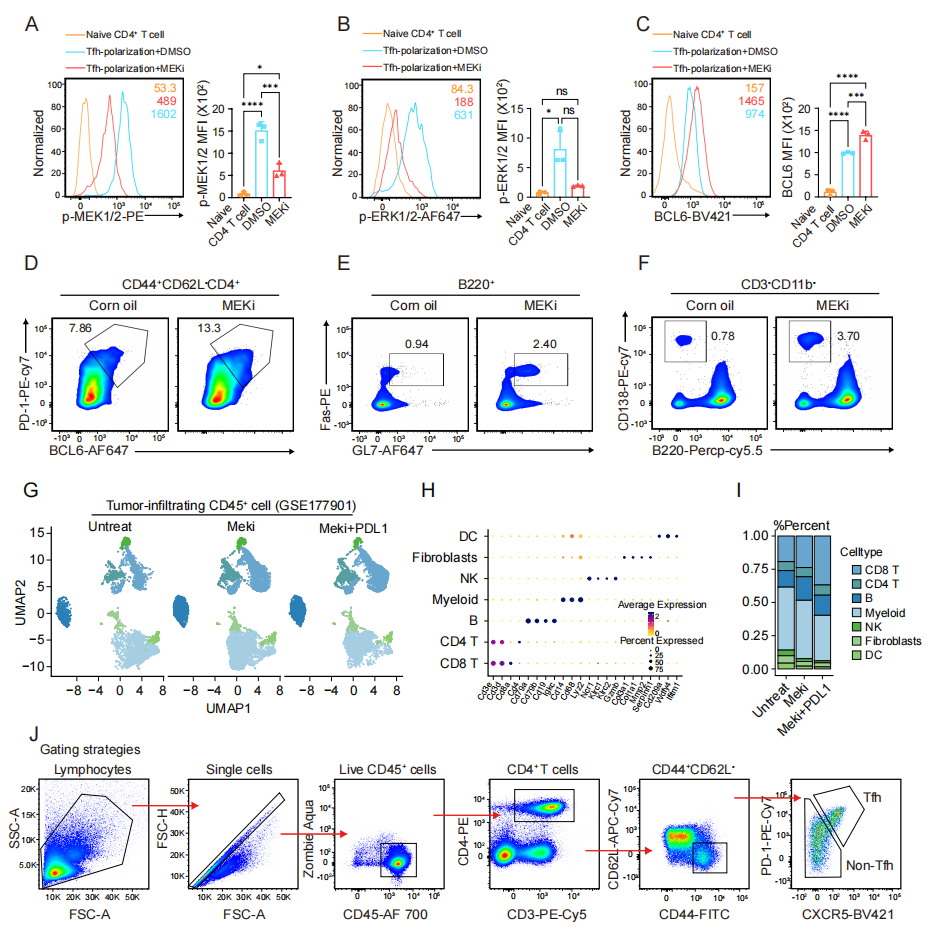


**Figure S4.** MEK1/2 inhibitor enhances Tfh development in SRBC immunization model and murine melanoma model, related to Figure 6 and Figure 7.

(A-C) Representative histograms of p-MEK1/2, p-ERK1/2 and BCL6 expression in Naïve CD4^+^ T cell group, DMSO group and MEKi group. MFI is summarized on right (n=3). (D) Representative psuedocolor plots showing the percentages of BCL6^+^PD-1^+^ among CD44^+^CD62L^-^CD4^+^ cells from SRBC immunized B6 mice with trametinib or corn oil treatment at day 7. (E) Representative psuedocolor plots showing the percentages of Fas^+^GL7^+^cells among B220^+^cells from SRBC immunized B6 mice with trametinib or corn oil treatment at day7. (F) Representative psuedocolor plots showing the percentages of B220^-^CD138^+^cells among CD3^-^CD11b^-^cells from SRBC immunized B6 mice with trametinib or Corn oil treatment at day7. (G) UMAP plot showing the dynamic change of 7 sub-clusters from tumor-infiltrating CD45^+^ immune cells among untreat, MEKi treatment and MEKi plus anti-PD-L1 treatment. (H) Dot plot showing the relative average expression of cluster-defining signature genes (x axis) across the clusters (y axis). (I) Stacked barplot showing the percentages of each sub-clusters from CD45^+^ immune cells among untreat , MEKi treatment and MEKi plus anti-PD-L1 treatment, related to the UMAP plot in (H). (J) Gating strategy for identification of Tfh (CXCR5^+^PD-1^+^) cells in dLNs of B16F10 tumor-bearing mice at day 15. Data were representative of at least three independent experiments. Error bars show Mean ± SD; P values in (A-C) were calculated using one-way ANOVA followed by Tukey’s multiple-comparisons test; *, P < 0.05; **, P < 0.01; ***, P < 0.001; ****, P < 0.0001; ns, no significance.


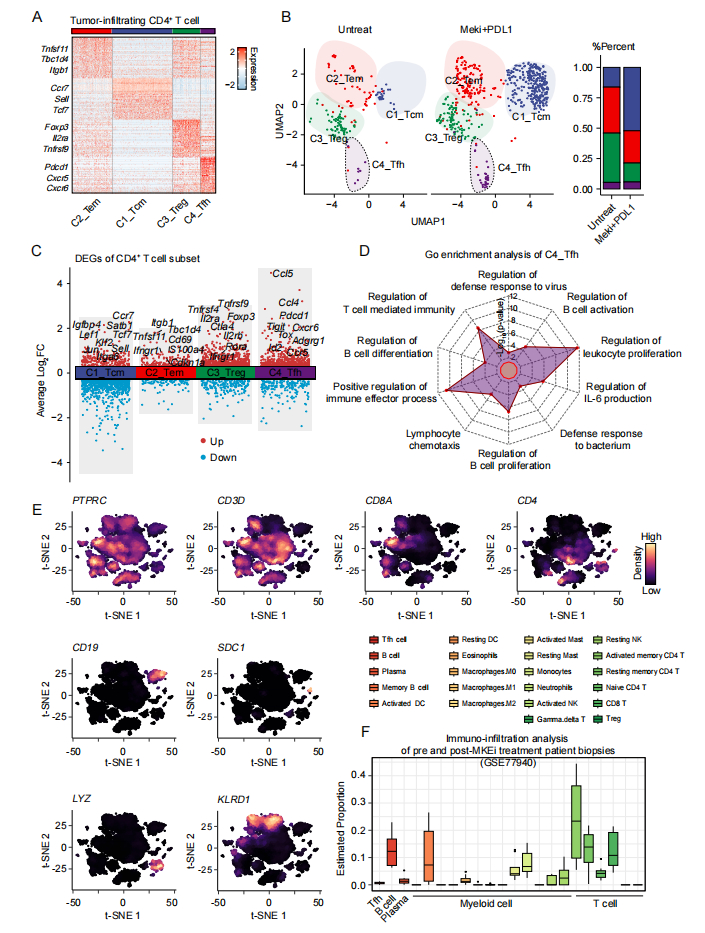


**Figure S5.** Additional details of the bioinformatics analysis in murine and patient melanoma data, related to Figure 7 and Figure 8.

(A) Heatmap showing scaled expression of differentially expressed genes(rows) among different CD4^+^ Tcell subpopulations (columns), representative signature genes of each cluster were highlighted (left). (B) UMAP plot showing the dynamic change of 4 sub-clusters from tumor-infiltrating CD4^+^T between untreat and MEKi plus anti-PD-L1 treatment. Stacked barplot summarizing the percentages of each sub-clusters on right. (C) Differential gene expression (DEGs) analysis showing up- and down-regulated genes across 4 sub-clusters from tumor-infiltrating CD4^+^T cells between untreat and MEKi treatment, representative signature genes of each cluster were highlighted. (D) Radar plot showing the degree of enrichment of representative pathways for the up-upregulated gene identified from tumor-infiltrating C4_Tfh cluster using enrichment analysis. (E) UMAP density plot showing the expression of cluster-defining signature genes of tumor-infiltrating CD45^+^ immune cells from the biopsy of melanoma patients. (F) Box plot showing the estimated proportion of all infiltrating immune cell types in biopsies from tumor patients.


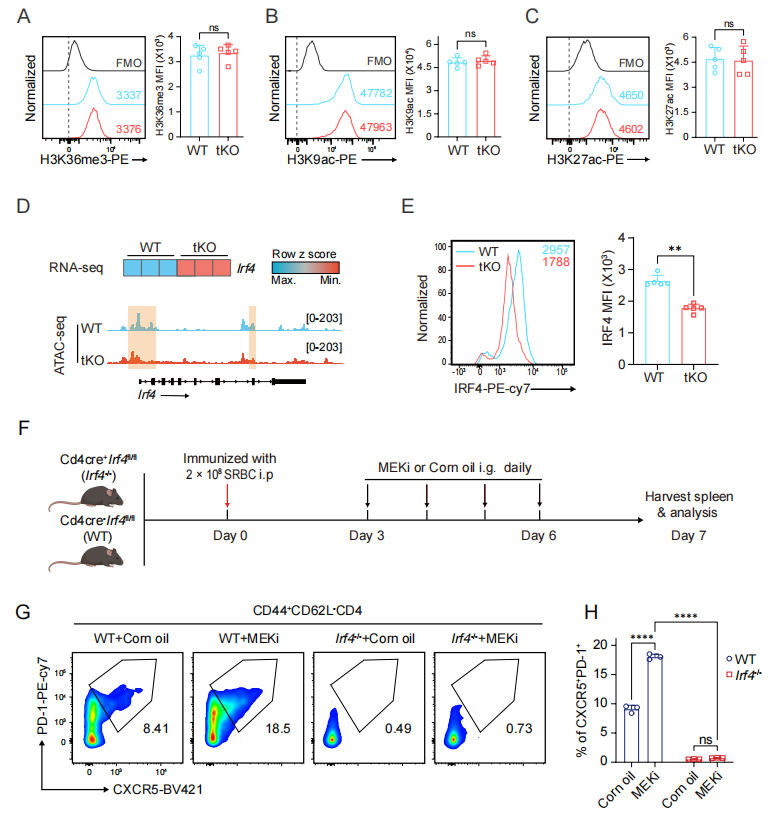


**Figure S6.** Additional details of the FCM analysis data and IRF4 loss-of-function experiments, related to Figure 4 and Figure 5.

(A-C) Representative FCM histograms (left panel) of H3K36me3, H3K9ac,and H3K27ac MFIs on transferred CD45.2^+^ WT or *Mek*1/2-tKO OT-II T cells in dLNs from recipient mice on day 7 post-immunization, MFI were depicted alongside (n=5). (D) Heatmap displaying the relative expression of *Irf4* characterized by RNA-seq and IGV showing representative ATAC-seq signal tracks at the *Irf4* locus from *Mek*1/2-tKO versus WT CD45.2^+^OT-II T cells on day 7 post OVA/CFA immunization. (E) Representative histograms and bar plots of IRF4 expression in CXCR5^+^PD-1^+^ cells from *Mek*1/2-tKO or WT CD45.2^+^OT-II mice measured by FCM on day 7 after OVA/CFA immunization (n=5). (F) Schematic of IRF4 loss-of-function experiments. (G-H) Representative psuedocolor plots showing the percentages of CXCR5^+^PD-1^+^ cells among CD44^+^CD62L^-^CD4^+^ cells from SRBC-immunized *Cd4*-Cre^+^*Irf4*^fl/fl^ or *Cd4*-Cre^-^*Irf4*^fl/fl^ mice treated with trametinib or corn oil on day 7 (n=3). The data are representative of at least three independent experiments. The error bars represent the means ± SD; P values in (A-C) were calculated using an unpaired two-tailed t test; P values in (E) were calculated using mann-whitney u test; P value in (H) was calculated using two-way ANOVA with Šídák's multiple comparison; *, P < 0.05; **, P < 0.01; ***, P < 0.001; ****, P < 0.0001; ns, not significant.


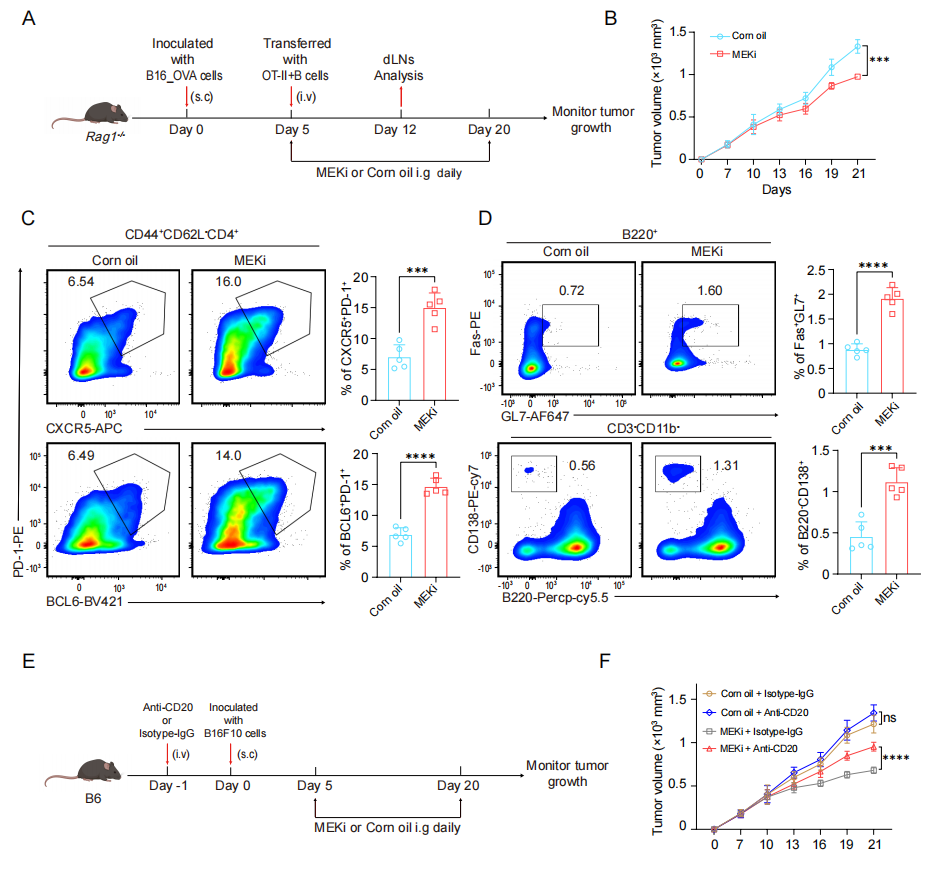


**Figure S7.** The antitumor efficacy of MEK inhibitors depend at least in part on their ability to promote humoral immune responses, related to Figure 7.

(A) Schematic of the tumor-bearing mouse experiments of OT-II and B cell transfer. (B) Growth

curves of B16_OVA melanoma in *Rag1*^-/-^ mice that underwent the adoptive transfer of OT-II and B cell cells. (C) Representative psuedocolor plots showing the percentages of CXCR5^+^PD-1^+^ and BCL6^+^PD-1^+^ cells among CD44^+^CD62L^-^CD4^+^ cells from recipient mice in the dLNs on day 12 after B16_OVA inoculation, bar plots were depicted alongside (n=5). (D) Representative psuedocolor plots showing the percentages of Fas^+^GL7^+^ cells among B220^+^ cells and B220^-^CD138^+^ cells among CD3^-^CD11b^-^ cells from recipient mice in the dLNs on day 12 after B16_OVA inoculation, bar plots were depicted alongside (n=5). (E) Schematic of B-cell depletion experiments in tumor-bearing mice. (F) Growth curves of B16F10 melanoma in B6 mice that underwent the B-cell depletion.The data are representative of at least three independent experiments. The error bars represent the means ± SD; P values in (C-D) were calculated using unpaired two-tailed t test; P value in (B-F) was calculated using two-way ANOVA with Šídák's multiple comparison; *, P < 0.05; **, P < 0.01; ***, P < 0.001; ****, P < 0.0001; ns, not significant.
